# Ribonucleotides Amplify Mitochondrial DNA‐Driven cGAS–STING Activation via FdUMP‐ribonucleotide Hybrid Particles to Potentiate Chemoimmunotherapy

**DOI:** 10.1002/advs.77614

**Published:** 2026-09-21

**Authors:** Chao Wang, Chenxuan Zhao, Yesong Yao, Zhiwen Zou, Anxin Shi, Zhuo Feng, Mingkang Yang, Yue Yu, Yang Liu, Xuehui Rui, Jinhui Wu

**Affiliations:** ^1^ State Key Laboratory of Pharmaceutical Biotechnology, Affiliated Drum Tower Hospital, Medical School of Nanjing University Nanjing University Nanjing China; ^2^ Medical 3D Printing Center, Orthopaedic Institute, Department of Orthopaedic Surgery, School of Basic Medical Sciences, The First Affiliated Hospital Suzhou Medical College Soochow University Suzhou China; ^3^ Institute of Energy Hefei Comprehensive National Science Center Hefei Anhui China; ^4^ Chemistry and Biomedicine Innovation Center Nanjing University Nanjing China; ^5^ Jiangsu Key Laboratory for Molecular Medicine Nanjing University Nanjing China; ^6^ Research Center for High Altitude Medicine Xining China; ^7^ Wuxi Xishan NJU Institute of Applied Biotechnology Wuxi China

**Keywords:** cancer research, chemistry, chemoimmunotherapy, downregulation and upregulation, interferon, intracellular, mitochondrial dna, nucleotide, stimulator of interferon genes

## Abstract

Intracellular nucleotide imbalance between ribonucleotides and deoxyribonucleotides can trigger cytosolic mitochondrial DNA (mtDNA) release and subsequent activation of the cGAS–STING pathway. However, fluoropyrimidine‐induced nucleotide imbalance is constrained by their metabolic instability. Here, we leverage ribonucleotides to amplify floxuridine (FUDR)‐induced mtDNA release and cGAS–STING activation for enhanced cancer chemoimmunotherapy. We identified that cytidine monophosphate (CMP) significantly enhances FUDR‐mediated activation of the mtDNA‐driven cGAS–STING pathway, inducing over 300‐fold upregulation of the interferon gene *Ifnb1* and initiating STING‐dependent antitumor immunity. Genome‐wide CRISPR‐Cas9 screening identified five pyrimidine‐metabolizing enzymes (CMPK1, CMPK2, TYMP, UPP1, and UPP2) that are responsible for the metabolic instability of floxuridine monophosphate (FdUMP). CMP and its intracellular metabolites can metabolically block these enzymes, thereby increasing the peak intracellular concentration of FdUMP by over 500‐fold and extending its half‐life by more than 20‐fold, which in turn promotes sustained intracellular nucleotide imbalance. To translate this mechanism into therapy, we co‐encapsulated FdUMP and CMP into hybrid lipid nanoparticles (FC‐NPs). FC‐NPs robustly activated STING‐dependent antitumor immunity, achieving remarkable therapeutic efficacy in multiple murine colorectal cancer (CRC) models. Our study not only addresses the long‐standing issue of fluoropyrimidine metabolic instability but also reveals a novel ribonucleotide‐mediated immunomodulatory mechanism, providing a promising strategy to potentiate chemoimmunotherapy in CRC.

## Graphical abstract

This work develops a metabolic sharing strategy that employs cytidine monophosphate (CMP) to competitively reprogram floxuridine (FUDR) pyrimidine metabolism in colorectal tumor cells. Elevated intracellular FdUMP induces nucleotide imbalance, drives mitochondrial DNA release and cGAS‐STING innate immune cascade activation, and potentiates robust antitumor chemoimmunotherapy with hybrid lipid nanoparticle (LNP) delivery.

## 1 Introduction

Nucleotides are the building blocks of nucleic acids and represent a pan‐cancer metabolic dependency. Indeed, to support their uncontrolled proliferation, chemotherapy resistance, immune evasion, and metastasis, cancer cells rely heavily on hyperactive nucleotide metabolism [1, 2], exhibiting markedly elevated levels of ribonucleotides and deoxyribonucleotides by 6–11‐fold and 1.25–5‐fold, respectively relative to normal proliferating cells [3, 4]. Notably, accumulating evidence has indicated that intracellular nucleotide imbalance between ribonucleotides and deoxyribonucleotides acts as a key mitochondrial stressor, which can induce mtDNA release into the cytosol [5, 6]. Cytosolic mtDNA is efficiently recognized by cyclic GMP‐AMP synthase (cGAS), and this recognition activates the stimulator of interferon genes (STING) signaling pathway, initiating the secretion of type I interferons (IFN‐I) and the expression of interferon‐stimulated genes (ISGs), recruiting and activating innate immune cells, and ultimately priming antitumor immune responses [7]. Despite recent studies confirming the immunomodulatory potential of nucleotide imbalance‐induced mtDNA release, how to actively and effectively break through the adaptive metabolic barrier of cancer cells, manipulate the imbalance between ribonucleotides and deoxyribonucleotides to reach the immunostimulatory threshold, and thereby enhance antitumor immunity, remains underexplored. This critical research gap highlights an unmet need at the intersection of nucleotide metabolism targeting, cancer cell adaptation to therapy resistance, and cancer immunotherapy.

Fluoropyrimidines are widely used in cancer treatment and serve as first‐line chemotherapy agents for colorectal cancer (CRC), CRC ranks among the top contributors to global cancer‐associated mortality, responsible for roughly 700 000 deaths each year [8, 9]. Among CRC patients, 70%–80% receive fluoropyrimidine‐based regimens (e.g., FOLFOX), which have significantly improved 2‐year survival rates [10, 11]. As classic antimetabolic chemotherapeutics, fluoropyrimidines inherently target nucleotide metabolism: they modulate intracellular nucleotide imbalance in cancer cells, thereby inhibiting proliferation, inducing apoptosis, and even activating cGAS–STING cascade‐mediated antitumor immune responses [12, 13, 14, 15]. However, the therapeutic efficacy of fluoropyrimidines is severely constrained by their inherent metabolic instability and competition from supraphysiological levels of endogenous nucleotides in cancer cells [16, 17, 18]. These factors lead to insufficient intracellular persistence of active drug metabolites and dose‐limiting toxicities. Despite decades of efforts to optimize fluoropyrimidine‐based therapy, including pharmacological inhibition of dihydropyrimidine dehydrogenase (DPD) and thymidine phosphorylase (TYMP), as well as the development of prodrugs such as capecitabine, clinical outcomes remain suboptimal, particularly in advanced CRC [19]. Given that cancer cells have established a hyperactive metabolic homeostasis of ribonucleotides and deoxyribonucleotides to support their malignant phenotypes, we hypothesize that actively leveraging the shared metabolic pathways of ribonucleotides and deoxyribonucleotides could enhance the metabolic stability of fluoropyrimidines in cancer cells. This strategy would amplify nucleotide imbalance‐induced cGAS–STING pathway, highlighting an unexploited opportunity to integrate metabolic modulation with immunostimulation for enhanced CRC chemoimmunotherapy.

**FIGURE 1:**
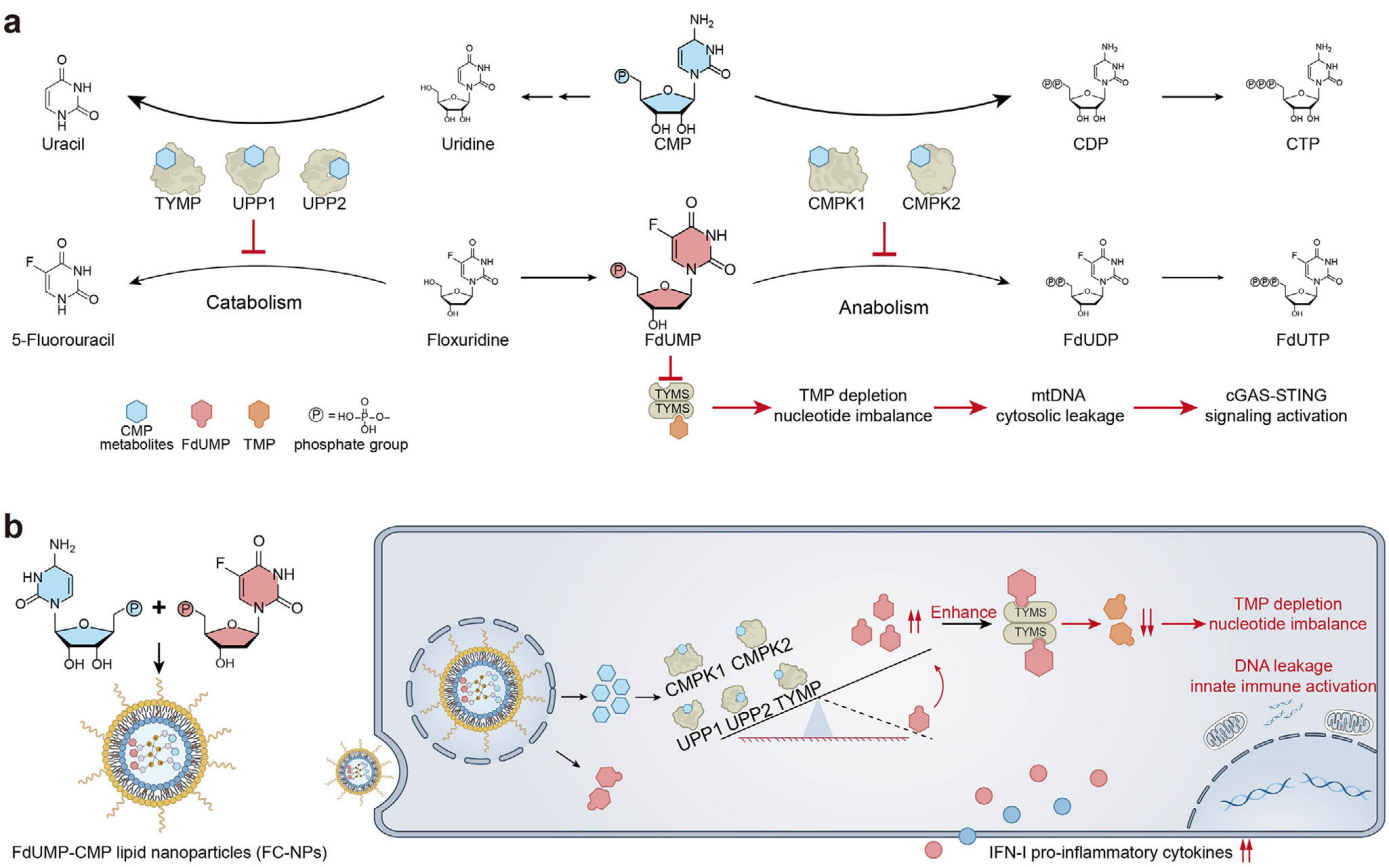
Shared metabolic strategy modulates intracellular floxuridine momophosphate metabolism to enhance therapeutic efficacy. (a) Intracellular CMP metabolites substitution for FUDR metabolism leads to the accumulation of FdUMP, depletion of TMP, and enhanced cytosolic DNA release induced cGAS–STING cascade signaling in cancer cells. CMP metabolites block catabolic and anabolic pathways of FdUMP to heighten intracellular FdUMP to inhibit the synthesis of TMP efficiently. UPP1, Uridine phosphorylase 1; UPP2, Uridine phosphorylase 2; TYMP, Thymidine phosphorylase; CMPK1, UMP‐CMP kinase; CMPK2, UMP‐CMP kinase 2 (mitochondrial) (b) Schematic illustrations of the lipid nanoparticles (FC‐NPs) co‐deliver CMP and FdUMP for enhanced antitumor efficacy.

Here, we explored a metabolic sharing strategy utilizing the naturally occurring, non‐toxic pyrimidine ribonucleotide cytidine monophosphate (CMP) to remodel floxuridine (FUDR) metabolism in tumor cells (Figure 1). Surprisingly, we found that CMP significantly enhances FUDR‐mediated activation of the mtDNA‐driven cGAS–STING pathway in cancer cells, inducing over 300‐fold upregulation of interferon‐β (IFN‐β) gene *Ifnb1* expression and initiating robust STING‐dependent antitumor immunity. Mechanistically, we identified five key enzymes (CMPK1, CMPK2, TYMP, UPP1, and UPP2) that constrain the therapeutic efficacy of FUDR. Competitive blockade of these enzymes by CMP and its intracellular metabolites rewires FUDR metabolism to promote robust FdUMP accumulation (Figure 1a). Specifically, CMP increased peak intracellular FdUMP levels by over 500‐fold and extended its half‐life by more than 20‐fold, thereby enhancing thymidylate synthase (TYMS) inhibition and promoting mtDNA release. This mtDNA release in turn synergistically activates cGAS–STING pathway and amplifies STING‐dependent antitumor immunity. To translate this insight into a therapeutic strategy, we engineered lipid nanoparticles co‐encapsulating FdUMP and CMP (FC‐NPs) (Figure 1b). FC‐NPs significantly enhance intracellular FdUMP persistence and therapeutic efficacy, amplify STING activation, and achieve potent tumor suppression in multiple CRC models. Our findings establish CMP as a clinically translatable metabolic modulator that overcomes the intracellular instability of FUDR, thereby potentiating both its cytotoxic and immunostimulatory effects. This work provides a rational framework for leveraging ribonucleotide metabolic modulation to enhance FUDR‐based chemoimmunotherapy.

## 2 Results

### 2.1 Ribonucleotides Amplify the Floxuridine‐Induced mtDNA‐Driven cGAS–STING Pathway

**FIGURE 2:**
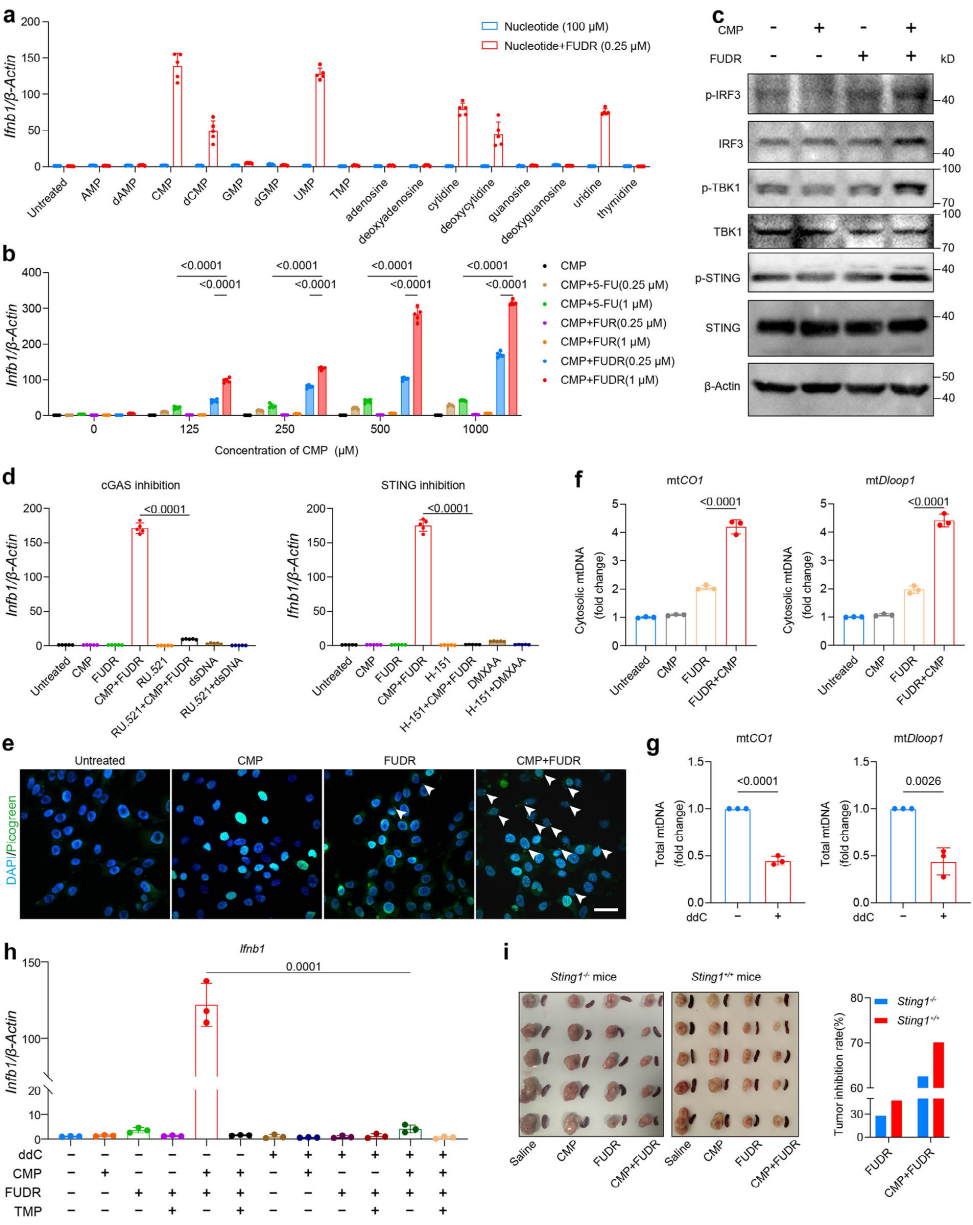
CMP promotes FUDR to activate the cGAS–STING signaling in cancer cells. (a) CT26 cells were treated in vitro with 5‐FU (left), FUR (middle), FUDR (right) or relevant vehicle controls for 24 h. The expression of *Ifnb1* was determined by qRT‐PCR (n = 5). (b) CT26 cells were treated with CMP, 5‐FU, FUR, FUDR or a combination thereof for 24 h in vitro. Expression of *Ifnb1* was determined by qRT‐PCR (n = 5). (c) For CT26 cells treated in vitro with CMP, FUDR, or their combination, Western blot analysis was performed using antibodies against β‐Actin (as loading controls), STING, phosphorylated STING (p‐STING), TBK1, phosphorylated TBK1 (p‐TBK1), IRF3, and phosphorylated IRF3 (p‐IRF3). (d) CT26 cells were pretreated with cGAS (left) and STING (right) inhibitor, then these cells and control CT26 cells were treated in vitro with CMP, FUDR, dsDNA (for cGAS activation), DMXAA (for STING activation) or their combinations respectively for 24 h in vitro. The expression of *Ifnb1* was determined by qRT‐PCR (n = 5). (e) Representative merge images of DAPI‐stained (blue) and PicoGreen‐stained (green) CT26 nuclei 24 h after indicated treatment. Arrowheads highlight cytosolic micronuclei (outside the nucleus). Scale bar, 50 µm. (f) CT26 cells were pretreated in vitro with CMP, FUDR or their combination respectively for 24 h, then the cytosolic contents without mitochondria of these cells were isolated wherein gene mtDNA *CO1* and *Dloop1* were determined by qPCR (n = 3). (g) Mitochondria were isolated from CT26 cells treated with 20 µM ddC for 9 days cells and total mtDNA levels analyzed by q‐PCR amplification of *CO1* (left) and *Dloop1* (right) in (n = 3). (h) CT26 cells were treated with ddC for 9 days to inhibit mtDNA synthesis, then these cells and control CT26 cells were treated in vitro with CMP, FUDR, TMP or their combinations respectively for 24 h. The expression of *Ifnb1* was determined by qRT‐PCR (n = 3). (i) MC38 tumor growth of WT C57BL/6 mice or *Sting1* ^−/−^ C57BL/6 mice. These mice were treated with Saline (n = 5), CMP (n = 5), FUDR (n = 5) or CMP+FUDR (n = 5). MC38 tumor weight at the endpoint with each dot representing a mouse. Pictures of MC38 tumors and spleens at the endpoint day 24. MC38 tumor inhibition rates were calculated at the endpoint day24. Data information: Error bars represent SD for all panels, and center values represent mean. Statistical significance was assessed using one‐way ANOVA with Turkey's multiple comparisons test (b, d, e, h); two‐tailed paired *t*‐test (g).

We were surprised to find that pyrimidine ribonucleotides, particularly CMP, can enhance FUDR‐mediated activation of the cGAS–STING cascade signaling in cancer cells, thereby boosting antitumor immune responses. When antimetabolic chemotherapies including fluoropyrimidines trigger genomic and mtDNA damage within tumor cells, fragmented DNA releases into the cytosol and is sensed by cGAS, which initiates the innate immune cGAS–STING signaling cascade in cancer cells [6, 7, 14]. Here, we first screened combinations of nucleotides with FUDR and found that pyrimidine ribonucleotides, especially CMP, significantly promoted FUDR‐mediated upregulation of *Ifnb1* transcriptional levels (Figure 2a). Meanwhile, we compared the transcriptional levels of *Ifnb1* induced by 5‐FU, FUR, and FUDR in the presence of CMP. FUDR produced the strongest stimulatory effect, driving *Ifnb1* transcription up more than 300‐fold (Figure 2b). However, CMP alone did not exert any stimulatory effect. Compared to FUDR, the stimulatory effect of CMP on 5‐FU and FUR was weak to negligible. Notably, the combination of CMP and FUDR exerted significantly stronger activation of the cGAS–STING cascade signaling than FUDR alone (Figure 2b,c).

To further explore the mechanism by which CMP amplifies FUDR‐mediated STING activation, we first used the cGAS inhibitor RU.521 [20] and the STING inhibitor H‐151 [21] for validation. We found that CMP+FUDR‐induced transcription of *Ifnb1* depended on both cGAS and STING (Figure 2d). We further examined cytosolic DNA release in cancer cells. FUDR‐induced cytosolic micronuclei in cancer cells was significantly amplified by CMP (Figure 2e). Compared with genomic DNA damage, the cytosolic mtDNA release shows a greater inducing effect on cGAS–STING cascade signaling [6]. Quantitative PCR assays of isolated cytosolic fractions revealed that CMP+FUDR potently increased the leakage rate of damaged mtDNA into the cytosol (Figure 2f). Therefore, we depleted mtDNA from cancer cells by incubating them with 2’, 3’‐dideoxycytidine (ddC) for 9 days (Figure 2g). The expression of *Ifnb1* and the interferon‐stimulated gene *Mx1* was significantly suppressed in mtDNA‐depleted cancer cells treated with CMP+FUDR (Figures 2h and S1). The mtDNA depletion did not completely abolish the activation effect of CMP+FUDR on the cGAS–STING cascade signaling in cancer cells.

Gene copy number analysis at different locations in the mtDNA genome directly confirmed that nucleotide imbalance can induce mtDNA replication stress. To directly assess changes in mtDNA replication homeostasis, we referenced previous research [5] and selected five representative genes (*mtDloop*, *mtCytb*, *mtNd4*, *mtCOX3*, and *mtNd1*) along the mitochondrial heavy chain replication origin (O_H_). We isolated mitochondrial fraction then used qPCR to detect the relative copy number changes of each gene after CMP+FUDR combined treatment. The results showed that after CMP+FUDR treatment, the mtDNA copy number exhibited a position‐dependent gradient decrease: regions closer to the O_H_ showed a relatively smaller decrease in gene copy number, while regions farther from the O_H_ showed a progressive decrease (Figure S2a). This gradient decrease in copy number along the replication direction is a typical molecular feature of replication fork arrest and replication process obstruction, indicating that CMP+FUDR‐induced pyrimidine nucleotide imbalance triggers mtDNA replication stress, which is the core inducing factor for subsequent mtDNA breakage and release.

Furthermore, exogenous TMP supplementation fully abrogated the upregulation of *Ifnb1* transcripts triggered by FUDR+CMP treatment (Figures 2g and S1). The FUDR‐induced *Ifnb1* transcript level gradually decreased with increasing TMP supplementation concentration (Figure S3). To further investigate this process, we verified that mtDNA release is a key factor in activating the cGAS–STING pathway through VDAC1 oligomerization blocking experiments, supplementing this with functional verification experiments using the VDAC1 inhibitor VBIT‐4. VDAC1 is a key channel protein located on the outer mitochondrial membrane that mediates the release of mtDNA fragments into the cytosol. Experimental results showed that inhibiting VDAC1 oligomerization with VBIT‐4 significantly suppressed CMP+FUDR‐induced *Ifnb1* transcriptional upregulation, with an inhibition level comparable to that of ddC‐induced mtDNA depletion (Figure 1h and S2b,c). This result further confirms that the release of mtDNA from mitochondria into the cytosol via VDAC1 after replication stress is a key factor in the activation of the cGAS–STING pathway in tumor cells by CMP+FUDR.

Next, we established MC38 xenografts with STING‐deficient (*Sting1* ^−/−^) C57BL/6 mice. Compared with wild type (WT) C57BL/6 mice, the inhibition rate induced by CMP+FUDR treatment of MC38 tumors was significantly reduced in *Sting1* ^−/−^ mice (Figures 2i and S4). This finding could be explained by the fact that the cytosolic mtDNA release‐activated cGAS–STING cascade signaling in cancer cells is essential for the induction of antitumor immunity [22, 23].

### 2.2 Ribonucleotides Potentiate Floxuridine Efficacy by Inducing Nucleotide Synthesis Imbalance

**FIGURE 3:**
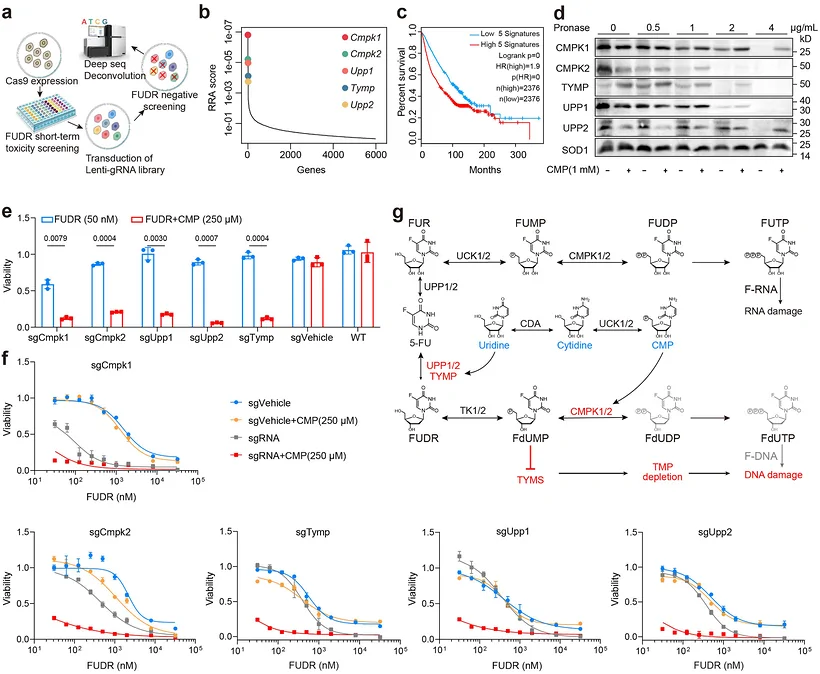
CMP potentiates the therapeutic activity of FUDR via competitive metabolism. (a) The main experimental procedures of the CRISPR‒Cas9‐based gRNA library negative screening for FUDR. (b) The top hits of pyrimidine metabolism‐associated genes in the CRISPR–Cas9‐based gRNA library negative screen were *Cmpk1*, *Cmpk2*, *Tymp*, *Upp1* and *Upp2*. Genes were ranked by the difference between their RRA (Robust Rank Aggregation) scores in the FUDR‐treated samples. (c) Kaplan‒Meier curves of overall survival of patients (n = 4752) with high (top quantile, red) or low (bottom quantile, blue) expression of 5 signatures (including *CMPK1, CMPK2, TYMP, UPP1* and *UPP2*). Low expression of 5 signatures predicted better survival than high expression of 5 signatures (p = 0). The data were isolated from The Cancer Genome Atlas (TCGA), and n is the number of biologically independent samples were plotted by GEPIA2.^48^ (d) DARTS analysis of CMPK1, CMPK2, TYMP, UPP1 and UPP2 stabilization in A549 lysates that were incubated with 1 mM CMP or vehicle for 30 min, followed by protein digestion at increasing pronase concentration and assessment of CMPK1, CMPK2, TYMP, UPP1 and UPP2 degradation by western blot. (e) Relative viability of CT26 cells, Cmpk1‐KO CT26 cells, Cmpk2‐KO CT26 cells, Tymp‐KO CT26 cells, Upp1‐KO CT26 cells and Upp2‐KO CT26 cells treated with FUDR (50 nM) or FUDR+CMP (250 µM) for 48 h. (f) Relative viability of CT26 cells, Cmpk1‐KO CT26 cells, Cmpk2‐KO CT26 cells, Tymp‐KO CT26 cells, Upp1‐KO CT26 cells and Upp2‐KO CT26 cells treated with gradient concentrations of FUDR or the combination of FUDR+CMP (250 µM) for 48 h. (g) 5‐FU anabolic pathway and the metabolic speculation that CMP can promote FdUMP accumulation. FUR and FUDR are two key products of the 5‐FU anabolic process. FUR mainly participates in RNA synthesis. FdUMP generated from FUDR inhibits TYMS‐mediated synthesis of TMP from dUMP. UPP1, Uridine phosphorylase 1; UPP2, Uridine phosphorylase 2; TYMP, Thymidine phosphorylase; UCK, Uridine‐cytidine kinase1/2; TK1, Thymidine kinase, cytosolic; TK2, Thymidine kinase 2 (mitochondrial); CMPK1, UMP‐CMP kinase; CMPK2, UMP‐CMP kinase 2 (mitochondrial); CDA, Cytidine deaminase. Data information: Error bars represent SD for all panels, and center values represent the mean. The survival analyses in the figure were performed by log‐rank (Mantel‒Cox) test (c). Statistical significance was assessed using two‐tailed unpaired Student's *t*‐test (e).

To further investigate the mechanism by which CMP enhances FUDR‐mediated activation of the innate cGAS–STING pathway in cancer cells, we referenced previous research [24] and performed genome‐wide negative‐selection CRISPR‒Cas9 library screen to identify key pyrimidine metabolic enzyme genes that weaken the therapeutic activity of FUDR (Figure 3a). The top 5 highest‐scoring pyrimidine metabolic enzyme genes identified in this screening were *Cmpk1, Cmpk2, Tymp, Upp1 and Upp2* (Figures 3b and S5). The Cancer Genome Atlas (TCGA) data analysis [25] showed that patients with low expression of the 5 signature genes (*CMPK1*, *CMPK2*, *TYMP*, *UPP1* and *UPP2*) had better survival outcomes than those with high expression (Figure 3c). These findings further suggest that targeting the FUDR metabolic pathway mediated by these 5 signature genes may serve as a strategy to enhance therapeutic efficacy.

We were surprised to observe that CMP supplementation could simultaneously target all 5 enzymes. Although CMP is theoretically considered a substrate only for CMPK1 and CMPK2 [26, 27]. To investigate potential interactions between CMP and these enzymes, we performed a drug affinity responsive target stability (DARTS) assay, a method that assesses the binding of small molecules to target proteins by measuring their protection from protease‐mediated degradation [28, 29]. This assay demonstrated that both CMP and uridine monophosphate (UMP) were capable of stabilizing all 5 proteins in cancer cells (Figures 3d and S6), indicating that CMP and its intracellular metabolites may inhibit the weakening effect of these 5 enzymes on FUDR therapeutic activity (Figure 3g).

To further investigate the roles of these enzymes in FUDR metabolism, we established single‐gene knockout CT26 cell lines for each of the 5 genes (Figure S7). We tested the effects of a low concentration of CMP (0.25 mM), which did not enhance FUDR cytotoxicity in wild‐type CT26 cells (WT). However, in all 5 knockout cell lines, low concentrations of CMP significantly potentiated FUDR therapeutic activity, suggesting that these 5 enzymes are essential for the ability of CMP to enhance the effects of FUDR (Figure 3e,f). Notably, *Cmpk1*‐deficient (*Cmpk1*‐KO) cells exhibited enhanced sensitivity to FUDR even in the absence of exogenous CMP, highlighting its central role in impairing FUDR therapeutic activity. The proteins encoded by these 5 genes mediate the metabolic process of FUDR and FdUMP (Figure 3g). These findings further validate that CMP and its intracellular metabolites competitively inhibit the 5 key metabolic enzymes (CMPK1, CMPK2, TYMP, UPP1, and UPP2), thereby rewiring FUDR metabolism to facilitate FdUMP accumulation.

**FIGURE 4:**
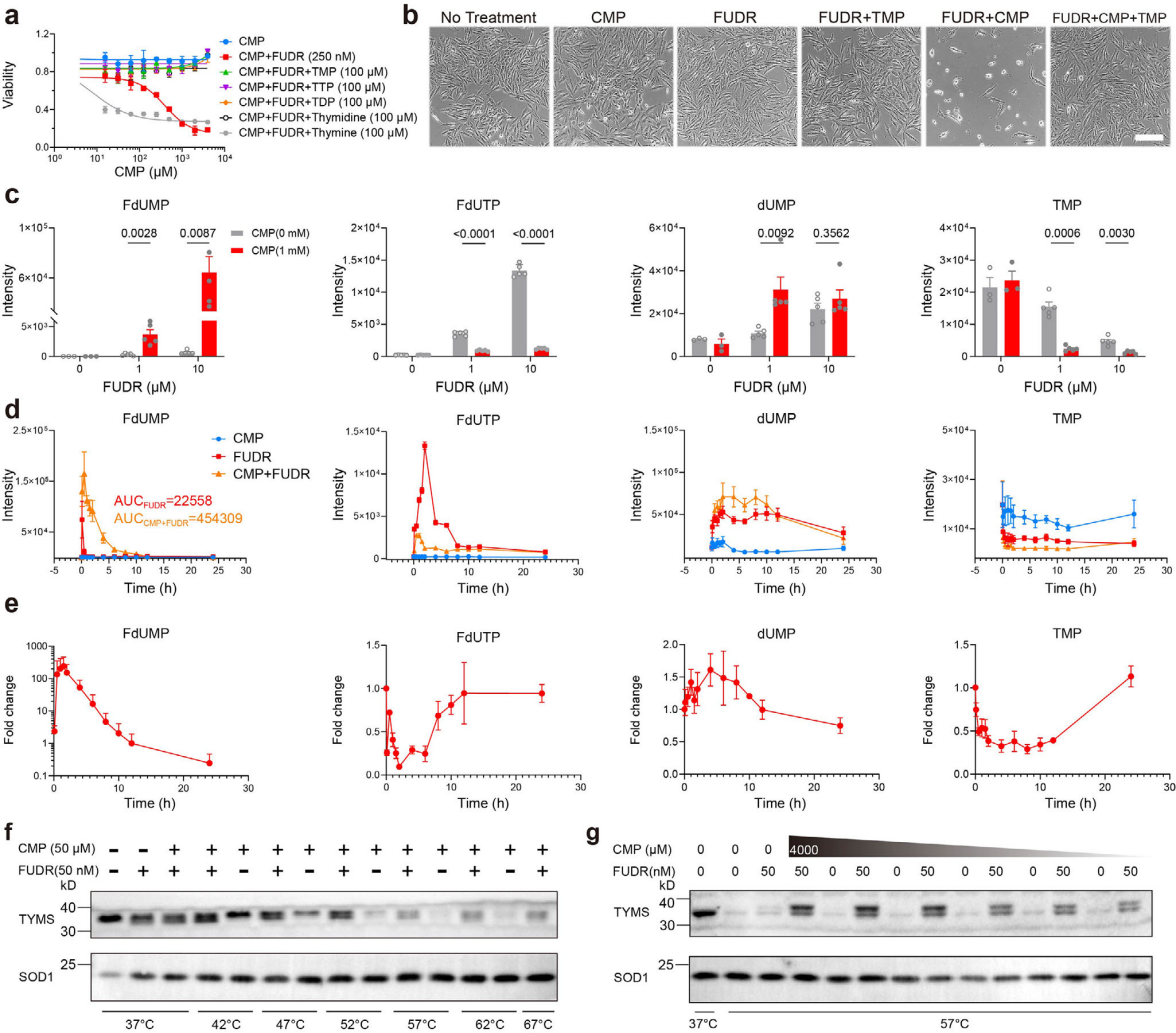
CMP heightens intracellular FdUMP to inhibit TMP synthesis. (a) Relative viability of CT26 cells treated with CMP, CMP+FUDR (250 nM) in the presence of 100 µM of the indicated thymine and its derivatives (n = 3). (b) Micrographs of A549 cells treated with CMP (1 mM), FUDR (250 nM), TMP (100 µM) or their combination for 4 days. Scalebar, 100 µm. (c) FdUMP, FdUTP, dUMP and TMP extracted from CT26 cells were determined via LC‐MS/MS. CT26 cells were treated with CMP, FUDR or their combination for 6 h (n = 3). (d) After treating CT26 cells with CMP, FUDR or their combination for the indicated times (0.1, 0.5, 1, 1.5, 2, 4, 8, 12, and 24 h), the content of FdUMP, FdUTP, dUMP and TMP extracted from CT26 cells were determined by LC‐MS/MS (n = 3). (e) The ratios of FdUMP, FdUTP, dUMP and TMP in CT26 cells treated with CMP+FUDR compared to those treated with FUDR (n = 3). (f) Representative western blots for the effects of 50 µM CMP‐assisted FUDR (50 nM) on thermal stabilization of TYMS protein. Protein levels were normalized against SOD1. CETSA was assayed in cell lysates. (g) 57°C CETSA analysis of TYMS stabilization in CT26 cells upon FUDR (50 nM) and gradient concentration of CMP treatment for 6 h. Protein levels were normalized against SOD1. Data information: Error bars represent SD for all panels except (c) where error bars represent s.e.m., and center values represent mean. Statistical significance was assessed using one‐way ANOVA with Turkey's multiple comparisons test (c).

To explore the potential mechanisms underlying the effects of CMP supplementation on FUDR treatment, we assessed whether CMP affects FUDR metabolism by determining its metabolites via liquid chromatography with tandem mass spectrometry (LC‐MS/MS). Even in the presence of CMP, supplemental TMP blocks the therapeutic activity of FUDR (Figure 4a,b). Supplementation with all thymine‐based nucleosides or nucleotides, except for thymine itself, reversed the synergistic antitumor activity of CMP and FUDR (Figure 4a), confirming a mechanism driven by TMP depletion rather than direct interference with DNA synthesis. Specifically, TMP supplementation strictly abolished FUDR activity (Figure 4a,b), as FUDR exerts its effects by inhibiting TYMS‐mediated TMP synthesis, and additional TMP could lead to the inactivation of FUDR. Therefore, we speculated that CMP does not directly interfere with the anabolism of TMP to enhance FUDR therapeutic activity. FUDR induces cancer cell death via TMP depletion. FUDR generates FdUMP via anabolism and FdUMP covalently binds and inhibits the TYMS‐mediated conversion of dUMP to TMP. CMP may alter the metabolic behavior of FUDR to amplify its therapeutic activity. In the presence of CMP, the levels of FdUMP and dUMP increased markedly, while the levels of FdUTP and TMP decreased (Figures 4c and S8). Compared with FUDR alone, the elimination half‐life of FdUMP increased 24‐fold, from 0.14 h to 3.45 h, in the presence of CMP, with a nearly 500‐fold increase in the peak concentration (Figure 4d,e). These results suggest that CMP supplementation enhances FdUMP accumulation and inhibits TYMS activity, thereby reducing TMP synthesis. Interestingly, the content of dUMP did not increase as significantly as that of FdUMP. Possibly, dUMP is involved in other metabolic pathways or is incorporated into DNA after anabolism.

To examine whether the presence of CMP enhances the inhibitory effect of FdUMP on TYMS, we performed a cellular thermal shift assay (CETSA) to assess drug–target engagement. In the presence of CMP, the stabilization of TYMS against thermal degradation induced by FUDR treatment was significantly enhanced (Figure 4f). FdUMP mediated TYMS stabilization against thermal degradation depended on the CMP concentration (Figure 4g). Similar effects were observed with FUDR analogs FTDR and FCDR (Figure S9), suggesting that CMP enhances the interaction of FUDR metabolites FdUMP with TYMS. These findings suggest that CMP supplementation enhances the interaction of FdUMP with TYMS, promoting TYMS stabilization and potentially improving the therapeutic efficacy of FUDR.

Given that CMP may acts as a metabolic sharer for driving the accumulation of FdUMP through shared 5 enzymes (CMPK1, CMPK2, TYMP, UPP1 and UPP2). We further performed LC‐MS/MS validation and confirmed that CMP treatment significantly increased intracellular levels of uridine (Figure S10a) and 2’‐deoxyuridine (Figure S10b) in CT26 cells, with this metabolic perturbation evident as early as 1 h post‐treatment. Consistent with these metabolic alterations, our DARTs assays (Figures 3d and S6) demonstrated that CMP and its metabolites stabilize 5 pyrimidine metabolic enzymes (CMPK1, CMPK2, TYMP, UPP1, and UPP2). Such stabilization enables competitive metabolic rewiring of FUDR catabolism, thereby amplifying the inhibitory impact of FdUMP on TYMS activity. To further characterize the metabolic changes induced by the 5 enzymes, we analyzed the intracellular levels of FdUMP metabolites in the corresponding gene‐knockout CT26 cancer cells. The combination of CMP (0.25 mM) and FUDR failed to increase FdUMP accumulation in CT26‐WT cells; however, this combination significantly elevated FdUMP levels in all 5 genes KO CT26 cells (Figure S11). In parallel, all these 5 genes KO cells also showed reduced levels of TMP. Consistently, genetic knockout of each individual enzyme further validated this regulatory axis. CMP treatment still effectively promoted FdUMP accumulation and TMP depletion in all five knockout cell lines, collectively demonstrating that these 5 enzymes are indispensable for CMP‐mediated FUDR sensitization.

**FIGURE 5:**
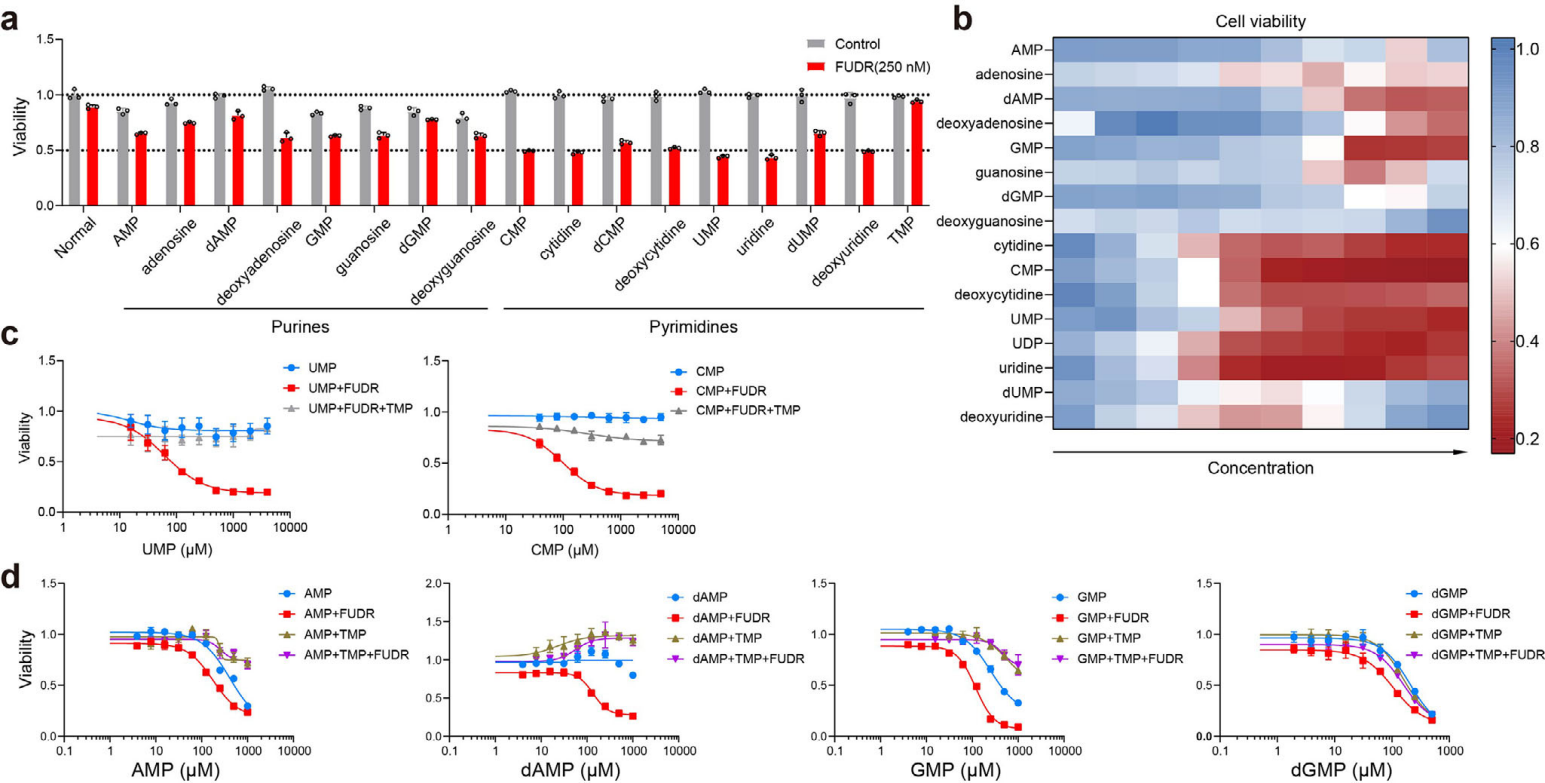
Pyrimidines, but not purines, potentiate FUDR therapeutic activity. (a) Purines and pyrimidines screening for which could enhance the sensitivity of CT26 cells to FUDR. Pyrimidines, but not purines, can potentiate the therapeutic activity of FUDR. (b) Relative changes in cell growth inhibition after treatment of cells with different purines or pyrimidines in combination with FUDR for 48 h. (c) Pyrimidines have synergistic effect with FUDR. Relative viability of CT26 cells treated with UMP, UMP+FUDR (250 nM), CMP and CMP+FUDR (250 nM) in the presence of 100 µM of TMP (n = 3). (d) Purines have strong cytotoxicity and has no synergistic effect with FUDR. Relative viability of CT26 cells treated with AMP, GMP, dAMP, dGMP, AMP+FUDR (250 nM), GMP+FUDR (250 nM), dAMP+FUDR (250 nM), dGMP+FUDR (250 nM) in the presence of 100 µM of TMP (n = 3). Data information: Error bars represent SD for all panels and center values represent mean.

We aim to verify whether other nucleotides can enhance the therapeutic activity of FUDR by examining the substrate levels of these nucleotide‐related metabolic enzymes. We wonder what the metabolic sharing pattern with FUDR is and whether there are other nucleotides that can enhance FUDR therapeutic activity. We further expanded the scope of nucleoside and nucleotide screening. Our results demonstrate that pyrimidines, but not purines, efficiently potentiated FUDR (Figure 5a,b). Previous studies revealed that purine nucleotides display cytotoxic effects at elevated concentrations [30] and may not be suitable for synergizing with FUDR (Figure 5d). In contrast, pyrimidine nucleotides such as CMP and UMP are well tolerated by cancer cells. High concentrations of CMP or UMP significantly amplified the therapeutic activity of FUDR, but additional TMP supplementation abolished this effect (Figure 5c). This means that CMP and UMP enhance the therapeutic activity of FUDR by enhancing the inhibitory potency of FdUMP on TYMS‐mediated TMP synthesis.

**FIGURE 6:**
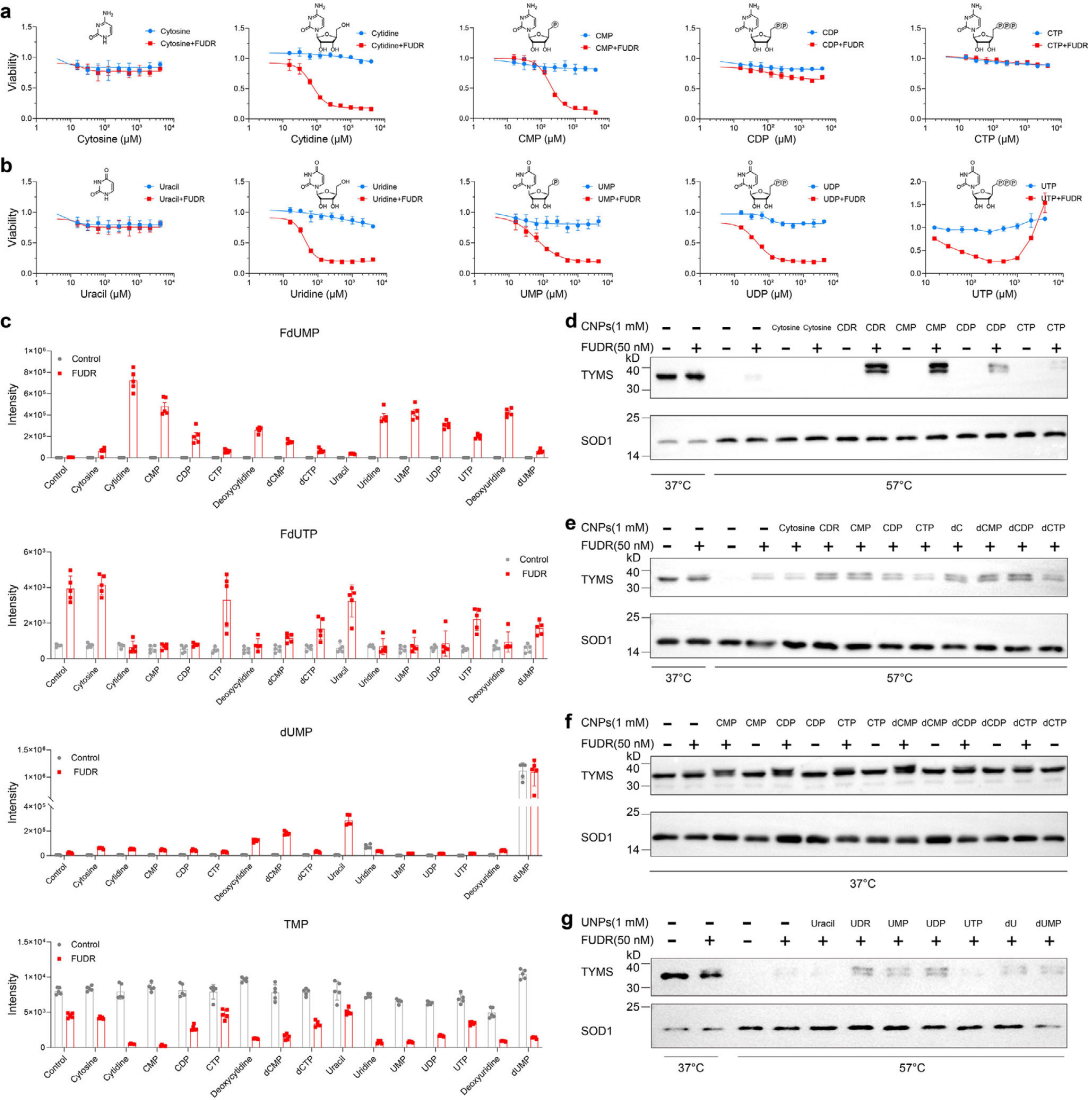
Pyrimidines Synergistically Block FdUMP Metabolism to Inhibit TMP Synthesis. (a, b) Screening for Pyrimidine Nucleotides Combined With FUDR. Relative Viability of CT26 Cells Treated With FUDR (250 nM) + Cytosine, Cytidine, CMP, CDP, CTP, Uracil, Uridine, UMP, UDP, and UTP for 48 h. (c) LC‐MS/MS for Determining FdUMP, FdUTP, dUMP and TMP Intensity in WT CT26 Cells Treated With FUDR (1 µM) or FUDR + Indicated Pyrimidine Nucleotides (1 mM) for 2 h. (d) 57°C CETSA Analysis of TYMS Stabilization in CT26 Cells Upon FUDR (50 nM) or FUDR + Indicated Cytosine Nucleotides Treatment for 6 h. Protein Levels Were Normalized Against SOD1. (e) 57°C CETSA Analysis of TYMS Stabilization in CT26 Cells Upon FUDR + Indicated Cytosine Nucleotides and Cytosine Deoxynucleotides Treatment for 6 h. Protein Levels Were Normalized Against SOD1. (f) 37°C Control CETSA Analysis of TYMS Stabilization in CT26 Cells Upon FUDR + Indicated Cytosine Nucleotides and Cytosine Deoxynucleotides Treatment for 6 h. Protein Levels Were Normalized Against SOD1. (g) 57°C CETSA Analysis of TYMS Stabilization in CT26 Cells Upon FUDR + Indicated Uracil Nucleotides and Uracil Deoxynucleotides Treatment for 6 h. Protein Levels Were Normalized Against SOD1. Data Information: Error Bars Represent SD for all Panels and Centre Values Represent Mean.

Next, we assessed whether cytosine‐ and uracil‐based nucleotide substrates of these 5 enzymes (CMPK1, CMPK2, TYMP, UPP1 and UPP2) exhibited synergy with FUDR. In terms of cell viability, cytosine, uracil, CDP, CTP, and UTP exerted no synergistic effect when combined with FUDR, while cytidine, uridine, CMP, UMP and UDP exerted a synergistic effect with FUDR (Figure 6a,b). In terms of intracellular nucleotides metabolism, we observed that (deoxy)cytidine, (d)CMP, (deoxy)uridine, (d)UMP, etc., as substrates for these 5 enzymes, promoted the accumulation of FdUMP and depletion of FdUTP and TMP (Figure 6c). Moreover, the results of CTSEA were highly consistent with the aforementioned results (Figure 6d–g). The substrates of these 5 enzymes all promoted FUDR‐mediated TYMS stabilization against thermal degradation.

**FIGURE 7:**
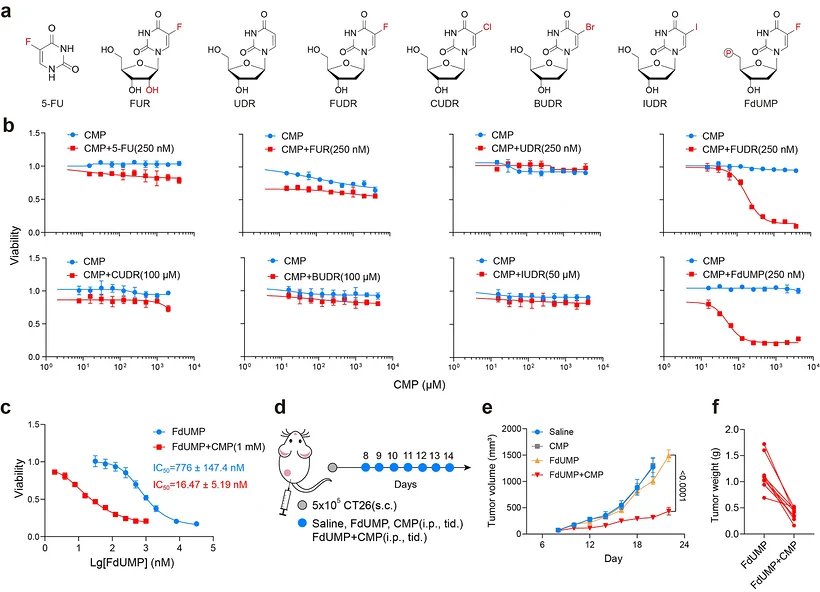
CMP augments the therapeutic activity of FdUMP. (a) Chemical structures of 5‐FU‐based chemotherapeutics. (b) Relative viability of CT26 cells treated with 5‐FU‐based chemotherapeutics in the presence of CMP at gradient concentrations versus CMP control (n = 3). (c) Relative viability of CT26 cells treated with gradient concentrations of FdUMP in the presence of CMP (1 mM) versus FdUMP alone (n = 3). (d) Schematics depicting the therapeutic schedule of CT26 colorectal cancer xenografts treated with intraperitoneal injection of FdUMP, CMP or FdUMP+CMP. i.p., intraperitoneal injection. tid., 3 times a day. (e) CT26 tumor growth curve of control BALB/c mice (n = 9), BALB/c mice treated with CMP (n = 9), BALB/c mice treated with FdUMP (n = 9), or BALB/c mice treated with CMP+FdUMP (n = 9). (f) CT26 tumor weight at the endpoint from (e), with each dot representing a mouse. Data information: Error bars represent SD for all panels except (i) where error bars represent s.e.m, and center values represent mean. Statistical significance was assessed using two‐way ANOVA with Tukey's multiple comparisons test (e).

We systematically screened 5‐FU‐based derivatives to further investigate the regulatory effects of CMP on FUDR and its metabolite FdUMP therapeutic efficacy (Figure 7a). 5‐FU is one of the most used broad‐spectrum antitumor drugs in clinic. A series of antimetabolite chemotherapeutics have been developed based on it. These drugs are structurally similar, with minor differences in substituent groups. Notably, only FUDR and its active metabolite FdUMP exhibited significant synergism with CMP, while 5‐FU and fluorouridine (FUR) showed no therapeutic enhancement (Figure 7b). This selective response can be attributed to fundamental differences in their metabolic activation pathways. Specifically, 5‐FU and FUR require conversion to FUDR via TYMP and UPP1/2 before being metabolized into FdUMP. However, CMP competes with these enzymes, inhibiting the conversion of 5‐FU and FUR to FUDR, which explains why CMP does not produce a synergistic effect with these two drugs (Figure 7b). In contrast, CMP metabolites competes with TYMP, UPP1/2, and CMPK1/2 to redirect FUDR metabolism, leading to FdUMP accumulation and generating a synergistic effect (Figure 7b). Further analysis revealed that CMP significantly amplified the therapeutic activity of FUDR in various mouse cancer cell lines (Figure S12a–d) and human cancer cell lines (Figure S12e–k), particularly in colorectal cancer.

We further verified the synergistic effect of CMP on FdUMP, the active metabolite of FUDR. CMP significantly amplified the therapeutic activity of FdUMP in vitro, showing a 37‐fold decrease in the half‐maximal inhibitory concentration (IC_50_) (Figure 7c). In CT26 xenograft models, CMP significantly enhanced the antitumor activity of FdUMP, despite the requirement for high‐frequency intraperitoneal administration (thrice daily for seven consecutive days) (Figures 7d–f and S13).

Collectively, we found that CMP acts as a metabolic sharer for FUDR and its active metabolite FdUMP. CMP promoted the therapeutic activity of FdUMP, not only in terms of toxicity to cancer cells but also in an increase in the activation effect of mtDNA release on innate immune cGAS–STING signaling. Clearly, these results indicate that CMP enhances the therapeutic activity of FdUMP by inhibiting TYMS mediated TMP synthesis. This finding further suggests that blocking the 5 signature gene‐mediated metabolism may be a strategy to enhance the therapeutic effects of FdUMP.

### 2.3 Lipid Nanoparticles Co‐Deliver Ribonucleotide and Floxuridine Monophosphate to Enhance Chemoimmunotherapeutic Efficacy

**FIGURE 8:**
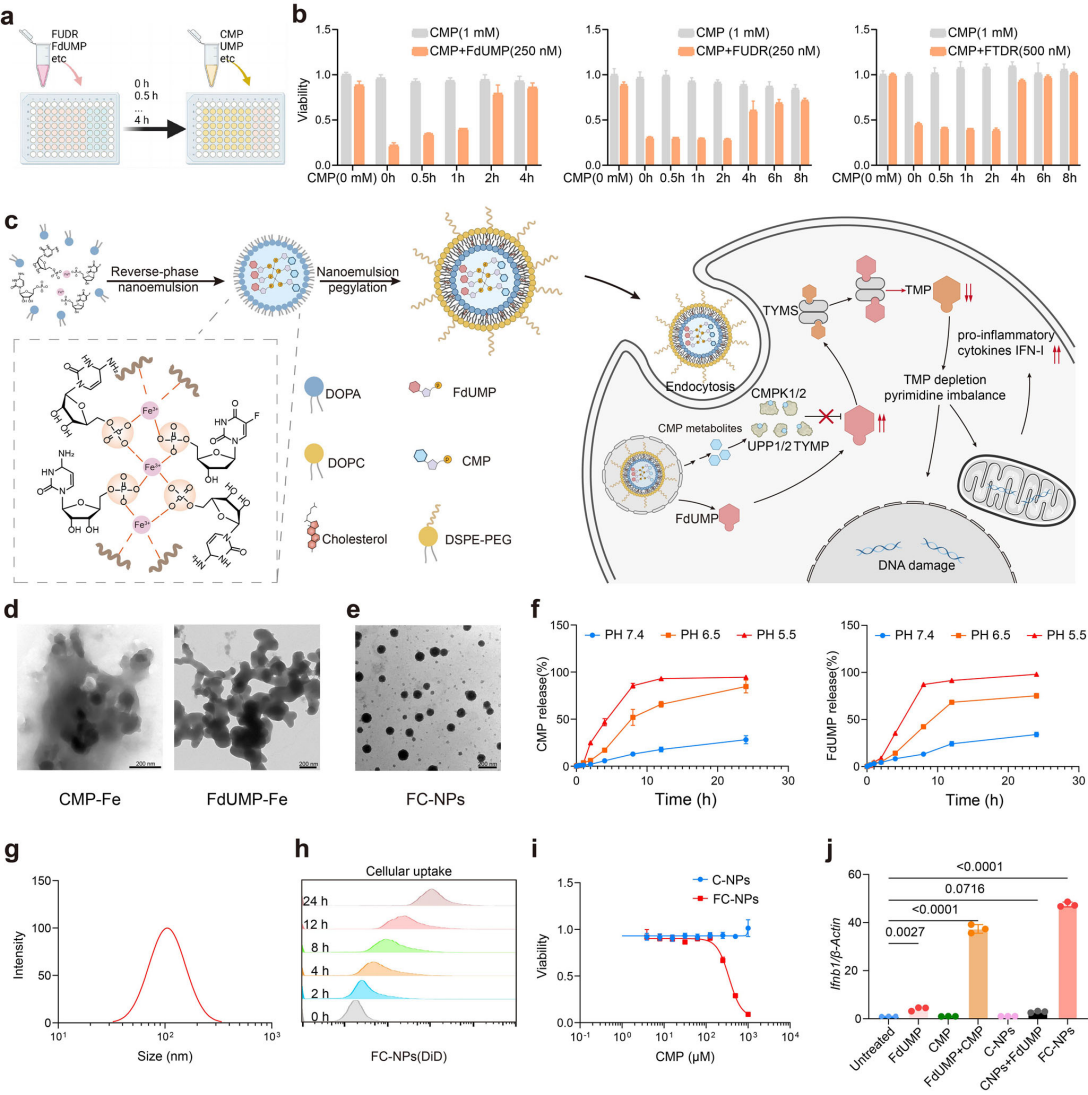
FC‐NPs synchronized delivery of CMP to enhance FdUMP therapeutic efficacy. (a) Scheme of the experiments for exploring the temporal patterns of the synergistic effects of CMP and pseudopyrimidine drugs such as FdUMP and FUDR. (b) Relative viability of CT26 cells treated with FdUMP (250 nM) + CMP (250 µM), FUDR (250 nM) + CMP (250 µM) or FTDR (500 nM) + CMP (250 µM) for 48 h. (c) Scheme of FC‐NPs co‐deliver CMP and FdUMP. The FC‐NPs is composed of FdUMP/CMP‐Fe coordination polymer, monosodium salt of 1,2‐dioleoyl‐sn‐glycero‐3‐phosphate (DOPA) and a PEG‐lipid layer with a 2:2:1 (m/m) mixture of 1,2‐dioleyl‐sn‐glycero‐3‐phosphocholine (DOPC), cholesterol and 1,2‐diastearoyl‐sn‐glycero‐3 ‐phosphoethanolamine‐N‐ [amino(polyethylene glycol)2000] (DSPE‐PEG2000). m/m, mass ratio. FC‐NPs boost the therapeutic activity of FdUMP: (1) the FC‐NPs promote the cellular uptake of CMP and FdUMP at the same time, and (2) CMP metabolites block multiple metabolic pathways of FdUMP to heighten intracellular FdUMP to inhibit the synthesis of TMP efficiently. IFN‐I, type I interferon; dNTP, deoxyribonucleoside triphosphates. (d) Self‐assembly behavior of CMP, FdUMP and Fe^3+^. Transmission electron microscopy (TEM) images of CMP‐Fe or FdUMP‐Fe coordination polymers formed by mixing CMP and FdUMP with Fe^3+^ (4:1, n/n) for 1 h. Scale bars, 200 nm; n/n, molar ratio. (e) TEM images showing homogeneous FC‐NPs formed by coating FdUMP/CMP–Fe coordination polymers with a PEGylated lipid layer. Scale bars, 200 nm. (f) The CMP (left) and FdUMP (right) release profiles from FC‐NPs at different pH values. (g) Dynamic light scattering analyses of FC‐NPs. (h) FC‐NPs increased the cellular uptake of CMP and FdUMP at the same time. CT26 cells were incubated with FC‐NPs for 2, 4, 8, 12 or 24 h, followed by analysis by flow cytometry. (i) Relative viability of CT26 cells treated with FC‐NPs or C‐NPs for 48 h. (j) CT26 cells were treated in vitro with FdUMP (250 nM), CMP (1 mM), CMP+FdUMP, C‐NPs, C‐NPs+FdUMP, FC‐NPs or untreated control for 24 h. The expression of Ifnb1 was determined by qRT‐PCR (n = 3). Data information: Error bars represent SD for all panels, and center values represent the mean. Statistical significance was assessed using one‐way ANOVA with Tukey's multiple comparisons test (j).

While the combination of CMP and FUDR (or FdUMP) shows efficacy, both CMP and FdUMP are highly hydrophilic molecules with low cell membrane permeability. Additionally, free drugs suffer from poor metabolic synchronicity and require high‐frequency administration (Figure 7d–f). More importantly, we observed that when FUDR or FdUMP was administered separately from CMP, the synergistic effect diminished with increasing dosing intervals beyond 2 h (Figure 8a,b). This indicates that the synergistic effect is dependent on the synchronous intracellular metabolism of CMP and FdUMP (or its prodrug FUDR). Such metabolic asynchrony likely explains why the combination of CMP and FdUMP was less effective in vivo than in vitro. To address these limitations, we developed a delivery system capable of co‐delivering FdUMP and CMP simultaneously, aiming for dose sparing and reduced side effects (Figure 8c). The co‐delivery of two or even multiple drugs can be achieved using nanodelivery system, an emerging and promising technology. CMP and FdUMP can theoretically be coordinated with metal ions to form nanoparticles by their phosphate groups. Through screening, we discovered that Fe^3+^ coordinated with various nucleotides, including CMP, UMP and FdUMP, coordinated their self‐assembly into coordination polymers with diameters ranging from nanometers to micrometers (Figure 8d and S14). Other divalent metal ions (Ca^2+^, Mn^2+^, Zn^2+^, Mg^2+^) failed to form stable nanoparticles under identical conditions, as confirmed by dynamic light scattering (data not shown).

Here, we referenced previous research [31, 32] and synthesized FdUMP/CMP‐Fe nanoparticles (FC‐NPs) and FdUMP/UMP‐Fe nanoparticles (FU‐NPs) via a two‐step method (Figure 8c). The FC‐NPs had a diameter of 106.8 nm (Figure 8e,g), and the FU‐NPs had a particle size of 122.4 nm (Figures S15 and S16). Both types of nanoparticles featured pH‐sensitive phosphate cores composed of FdUMP/CMP‐Fe or FdUMP/UMP‐Fe, which enabled pH‐responsive drug release (Figures 8f and S17), a highly desirable property for intracellular drug delivery. FC‐NPs and FU‐NPs prevented premature drug release in the bloodstream and protected FdUMP from enzymatic metabolism; however, they quickly released CMP, UMP and FdUMP in the acidic endosomal compartment and facilitated their endosomal escape via a proton sponge effect. We employed DiD, a fluorophore for labelling the lipid layer, to confirm the cellular uptake of FC‐NPs and FU‐NPs by CT26 cancer cells (Figures 8h and S18). Synchronous co‐delivery of FdUMP and CMP (or UMP) by FC‐NPs (or FU‐NPs) was essential for therapeutic activity. This was demonstrated by the lack of therapeutic effect observed with physical mixtures of blank nanoparticles (C‐NPs or U‐NPs) and free FdUMP administered concurrently (Figure 8i; Figures S19 and S20). Both FC‐NPs and FU‐NPs also induced robust and comparable levels of *Ifnb1* transcription in CT26 cells (Figures 8j and S21).

**FIGURE 9:**
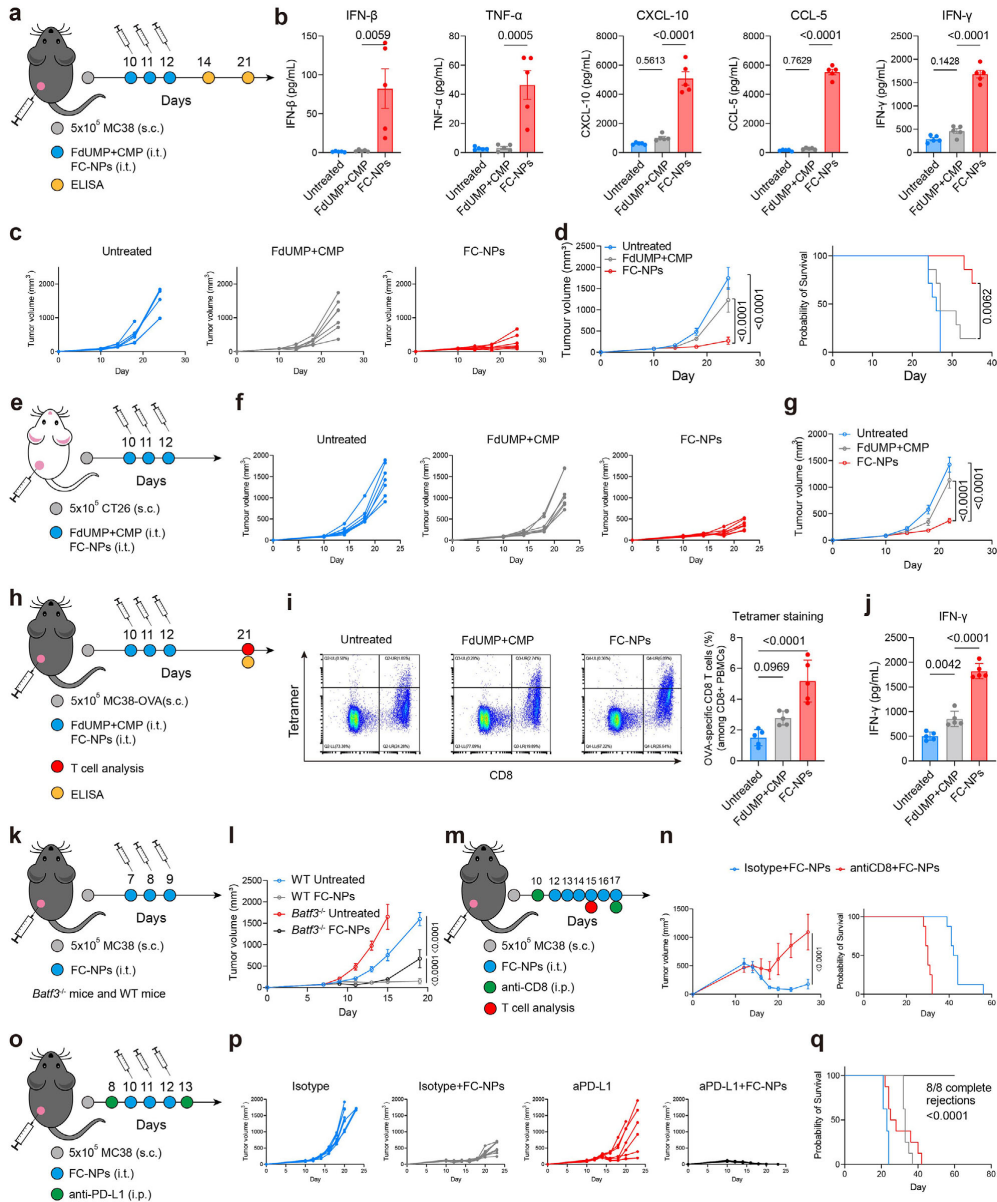
FC‐NPs boost chemo‐immunotherapy. (a) Schematics depicting the therapeutic schedule and immunoassay of MC38 cancer xenografts treated with intratumoral injection of FdUMP+CMP or FC‐NPs. (b) FC‐NPs treatment significantly increased the secretion of inflammatory cytokines. Serum cytokines (IFN‐β, TNF‐α, CXCL‐10, CCL‐5) and intratumoral IFN‐γ were measured by ELISA on day 14 and day 21 after the third dose, respectively. (c) Growth curves of MC38 tumors in individual mice treated with intratumoral injection of FdUMP+CMP or FC‐NPs or untreated. (d) FC‐NPs inhibited the growth of MC38 tumors and prolonged the survival of mice. Average tumor‐growth curves of MC38 tumors and survival curves of the mice in (a) (n = 7). (e) Schematics depicting the therapeutic schedule of CT26 cancer xenografts treated with intratumoral injection of FdUMP+CMP or FC‐NPs. (f) Growth curves of CT26 tumors in individual mice treated with intratumoral injection of FdUMP+CMP or FC‐NPs or untreated (n = 7). (g) FC‐NPs inhibited the growth of CT26 tumors. Average tumor‐growth curves of CT26 tumors of the mice in (f). (h) Schematics depicting the therapeutic schedule, T cell analysis and immunoassay of MC38‐OVA cancer xenografts treated with intratumoral injection of FdUMP+CMP or FC‐NPs. (i) The ratio of antigen‐specific T cells in PBMCs was significantly increased after FC‐NPs treatment. Representative scatter plots for OVA tetramer+ CD8 T cells and statistical analysis of the OVA tetramer+ CD8 T cells in peripheral blood T cells. The antigen‐specific T cell response in PBMCs was analyzed by OVA tetramer staining on day 21. (j) Interferon‐γ was increased in MC38‐OVA tumors after FC‐NPs treatment. Intratumoral IFN‐γ was measured by ELISA on day 21 after the third dose. (k) Schematics depicting the therapeutic schedule of MC38 cancer xenografts in *Batf3^−/−^* mice and WT mice treated with intratumoral injection of FC‐NPs or untreated. (l) The therapeutic activity of FC‐NPs on MC38 tumors was impaired in *Batf3* ^−/−^ mice. Average tumor‐growth curves of MC38 tumors of the mice in (k) (n = 8). (m) Schematics depicting the therapeutic schedule of MC38 cancer xenografts treated with intraperitoneal injection of anti‐CD8α antibody (aCD8) or isotype control antibody (Isotype) and intratumoral injection of FC‐NPs. (n) The therapeutic effect of FC‐NPs on MC38 tumors was severely impaired by CD8^+^ T cell depletion. Average tumor‐growth curves of MC38 tumors of the mice in (m) (n = 8). (o) Schematics depicting the therapeutic schedule of MC38 cancer xenografts treated with intraperitoneal injection of anti‐PD‐L1 antibody (aPD‐L1) or isotype control antibody (Isotype) and a combination of intratumoral injection of FC‐NPs. (p) The therapeutic effect of anti‐PD‐L1 on MC38 tumors was potentiated by FC‐NPs treatment. Growth curves of MC38 tumors in individual mice in (o) (n = 8). (q) The combination of anti‐PD‐L1+FC‐NPs caused mice to completely reject MC38 tumors and prolonged the survival of mice. Survival curves of the mice in (o). Data information: Error bars represent s.e.m. for all panels, and center values represent the mean. Statistical significance was assessed using one‐way ANOVA (b, i, j, n) or two‐way ANOVA (d, g, l,) with Bonferroni's multiple comparisons test, or log‐rank (Mantel‒Cox) test (d and q).

Next, we assessed the therapeutic efficacy of FC‐NPs and FU‐NPs in vivo using MC38 tumor‐bearing C57BL/6 mice. Mice were treated via intratumoral administration of FdUMP and CMP (or UMP) encapsulated in FC‐NPs (or FU‐NPs) on days 10, 11, and 12 post‐tumor inoculation (Figures 9a and S22). In contrast to previous experiments (Figures 7d–f and S4), where CMP and FUDR were daily administered in combination, we reduced the dosing frequency to three injections in this study to evaluate the dose‐sparing potential of FC‐NPs and FU‐NPs. Assessment of immune activation 2 days after the final injection (day 12) revealed significantly elevated levels of IFNβ and CXCL10 (measured at day 14) and TNFα, CCL5, and IFNγ (measured at day 21) in tumors treated with FC‐NPs or FU‐NPs compared to the corresponding free FdUMP+CMP or FdUMP+UMP admixtures (Figures 9b and S23).

Notably, FC‐NPs achieved 82% tumor growth inhibition in MC38 models, significantly outperforming the free FdUMP+CMP mixture (31%, p < 0.001; Figure 9c,d). FU‐NPs showed comparable efficacy (Figure S24). In addition, we performed the same evaluation in CT26 xenografts (Figures 9e and S25). Both FC‐NPs and FU‐NPs exhibited effective antitumor activity in the CT26 tumor model (Figures 9f,g and S26).

We further evaluated the ability of FC‐NPs and FU‐NPs to induce tumor antigen‐specific T cells using MC38‐OVA xenografts (Figures 9h and S27). Intratumoral injection of FC‐NPs (FU‐NPs) significantly increased the proportion of OVA antigen‐specific CD8^+^ T cells and the level of IFNγ in tumor compared with the free FdUMP+CMP (UMP) admixture (Figures 9i,j and S28). Therefore, the proportion of tumor antigen‐specific T cells in mice treated with FC‐NPs and FU‐NPs was significantly increased.

The critical role of adaptive antitumor immunity was underscored by two key experiments: First, the antitumor activities of FC‐NPs and FU‐NPs were significantly suppressed in *Batf3* ^−/−^ C57BL/6 mice deficient in cross‐presenting dendritic cells (Figures 9k,l and S29). This suggests that the efficacy depends on cross‐presentation of tumor antigens, essential for priming CD8^+^ T cell responses downstream of cGAS–STING pathway activation. Second, depletion of CD8^+^ T cells using anti‐CD8α antibodies significantly inhibited the antitumor efficacy of both nanoparticle formulations (Figures 9m,n and S30), confirming the indispensable role of CD8^+^ T cells for the efficacy of FC‐NPs and FU‐NPs.

When the cGAS–STING cascade signaling is activated, it induces the upregulation of PD‐L1 expression. This is negative feedback that follows the activation of adaptive immunity as activation of the innate immune system. Therefore, many preclinical and clinical studies usually combine ICB with agonists targeting STING [33, 34, 35]. Strikingly, combination therapy with anti‐PD‐L1 antibody and either FC‐NPs or FU‐NPs resulted in complete tumor rejection in all mice bearing MC38 tumors (Figures 9o–q and S31). In contrast, mice treated with anti‐PD‐L1 antibody, FC‐NPs or FU‐NPs alone failed to induce complete MC38 tumor regression.

Collectively, these findings establish FC‐NPs and FU‐NPs as effective platforms for synchronous drug delivery. And we demonstrate that FC‐NPs and FU‐NPs are potent inducers of antitumor immunity and can significantly enhance the efficacy of chemo‐immunotherapy, particularly achieving curative responses when combined with PD‐L1 blockade.

## 3 Discussion and Conclusion

Here, we propose a shared metabolic strategy by selecting natural ribonucleotides to compete with FUDR and FdUMP for shared 5 enzymes in cancer cells, thereby altering the metabolism of FdUMP for maximum nucleotide imbalance induced therapeutic efficacy. Taking the combination of FUDR and CMP as an example, this synergy is displayed in the more effective killing of cancer cells, while also significantly activating the intrinsic cGAS–STING cascade signaling of cancer cells to promote tumor immunogenicity. Through systematic combined screening of nucleotides and multiple 5‐FU‐based chemotherapeutics, we found that a variety of ribonucleotides can enhance FUDR. In the presence of cancer cell hypermetabolic activity, this strategy is inseparable from the synchronized metabolism of natural nucleotides and fluoropyrimidines. The FC‐NPs system we constructed using nucleotide coordination is expected to solve this problem.

The design of fluoropyrimidine drugs is based on structures similar to endogenous pyrimidine nucleotides. The problem here is that fluoropyrimidines will be metabolized by the shared metabolism pathways, leading to metabolic instability and insufficient target specificity. For instance, 5‐FU, a broad‐spectrum antitumor drug, has been used clinically for more than 60 years. However, the metabolic problems of 5‐FU still limit its clinical application. Although 5‐FU is well recognized as a TYMS‐targeting agent, its intracellular conversion to active FdUMP proceeds with low efficiency. Clinically, the original FOLFOX regimen has evolved into mFOLFOX6. This standard protocol mandates continuous 48‐h intravenous infusion of high‐dose 5‐FU (2 g/m^2^) combined with leucovorin to suppress de novo TMP synthesis, a therapeutic schedule linked to substantial systemic toxicities in patients. Our findings imply that pairing leucovorin with CMP‐modulated FUDR treatment may achieve equivalent anti‐tumor efficacy at substantially reduced drug dosages. Moreover, these metabolic distinctions also provide a mechanistic rationale explaining why CMP fails to potentiate the anti‐tumor activity of 5‐FU and FUR. A recent CETSA‐based study comparing intracellular metabolic signatures of 5‐FU, FUR, and FUDR further supports our findings [36]. That work defined distinct metabolic partitioning among these fluoropyrimidine analogs: FUDR preferentially enters deoxyribonucleotide metabolism, FUR primarily engages ribonucleotide metabolism, while 5‐FU participates in both pathways with a predominant preference for ribonucleotide metabolism. Medicinal chemists designed capecitabine as a prodrug of 5‐FU to overcome its metabolic problems. However, the prescription dose of capecitabine still reaches the gram scale. The S‐1 regimen is also designed to regulate the metabolism of 5‐FU in the gastrointestinal tract. In addition, trifluridine‐tipiracil tablets have been used to promote therapeutic activity by inhibiting the catabolism of trifluridine by TYMP. Researchers have been working hard to design various novel compounds to modulate the metabolism of 5‐FU, but this strategy is costly and has unpredictable side effects. At this time, the advantages of regulating the metabolic flux of fluoropyrimidines by using selected natural ribonucleotides are quickly revealed. Natural ribonucleotides do not require new molecular design.

We determined in detail the mechanism of synergy between CMP and FUDR. Notably, supplementation with TMP abolished this synergistic effect. LC‐MS/MS revealed that CMP significantly promoted the metabolic accumulation of FdUMP. FdUMP inhibition of TYMS blocks TMP synthesis, leading to thymineless death. Additionally, the resulting mtDNA release activates the intrinsic cGAS–STING cascade signaling in cancer cells. Through CRISPR‐Cas9 library screening, we found that 5 enzymes (CMPK1, CMPK2, TYMP, UPP1 and UPP2) impaired FUDR therapeutic activity. These 5 enzymes can be blocked by CMP and its intercellular metabolites uridine and 2’‐ deoxyuridine to amplify the therapeutic efficacy of FUDR. However, it is difficult to achieve the simultaneous inhibition of these 5 metabolic enzymes by developing small molecule inhibitors, which is costly and has unpredictable side effects. Here, we demonstrate the regulation of the metabolism of fluoropyrimidines by using selected natural ribonucleotides that do not require new molecular design, are inexpensive, and are easy to obtain.

In addition to FUDR and FdUMP, we also found that FCDR and FTDR nucleotide drugs therapeutic activity can be amplified through ribonucleotides (Figure S32). This requires further study of possible targets for this synergy. We found that CMP did not sensitize gemcitabine but dUMP did (Figure S33). This may be because CMP supplementation may increase the conversion of dCMP, which will dilute the intracellular production of gemcitabine monophosphate thus inhibiting the therapeutic effect of gemcitabine (Figures S33 and S34). However, dUMP is difficult to convert into dCMP, so it does not inhibit but rather enhances the therapeutic activity of gemcitabine. The same results have been verified with cytidine drugs such as decitabine and Ara‐C, which should be tested in future studies. This lays the foundation for the paradigm of manipulating the metabolic flux of pseudopyrimidine chemotherapeutic through selected natural nucleotides.

Moreover, we found that the activating effect of 5‐FU and FUR on cGAS–STING cascade signaling was significantly weaker than that of FUDR. This finding contrasts with recent studies that have revealed that 5‐FU can effectively activate the cGAS–STING cascade signaling in CT26 and MC38 cancer cells [14]. The anabolism of 5‐FU and FUR in cells mainly produces 5‐fluorouridine monophosphate (FUMP) to participate in RNA metabolism, while less FdUMP is produced, so the damaging effect of 5‐FU and FUR on DNA is weaker than FUDR (Figure 3g). These results also confirm previous studies. 5‐FU prefers to generate FUR rather than FUDR through intracellular anabolism [36]. Therefore, pyrimidine nucleotides such as CMP and UMP have minimum synergistic effects with 5‐FU. Combining 5‐FU with ICB represents an emerging strategy for chemo‐immunotherapy. However, our research results show that 5‐FU is not an ideal neoadjuvant for immunotherapyin this context, and the neglected FUDR may deserve more attention. The CMP co‐treatment strategy we describe is readily compatible with existing clinical regimens pairing 5‐FU or capecitabine with immune checkpoint blockade. By sustaining intratumoral FdUMP accumulation and amplifying immunogenic stress, this combinatorial approach holds promise for elevating therapeutic response rates and carries broad clinical applicability. Separately, FUDR represents an established standard agent for hepatic artery infusion chemotherapy (HAIC) in patients with colorectal cancer liver metastasis. CMP exhibited good biosafety in vivo (Figure S35). Moreover, local arterial delivery yields high intratumoral drug concentrations while minimizing systemic off‐target toxicity. Our CMP co‐administration strategy can be directly translated to this regional infusion setting, where concurrent local delivery of CMP may further amplify intratumoral anti‐tumor immune responses, offering a clear, actionable clinical translational path.

In the present study, we discovered that natural ribonucleotides can alter the metabolism of fluoropyrimidines, but this regulation method depends on metabolic competition within cancer cells. Only by using excessive amounts of natural ribonucleotides to compete with fluoropyrimidines for base‐nonspecific pyrimidine enzymes can more fluoropyrimidines accumulate and act on specific nucleotide metabolism targets, thereby exerting effective treatment. Therefore, we chose a shared metabolic strategy to manipulate the metabolism of fluoropyrimidines. Moreover, we systematically evaluated the therapeutic effects of FC‐NPs and FU‐NPs. We found that the therapeutic effects of FC‐NPs and FU‐NPs are inseparable from the adaptive immune system. Furthermore, both FC‐NPs and FU‐NPs exhibited powerful synergistic effects with ICB.

In conclusion, we describe a novel shared metabolic strategy by selecting natural ribonucleotides to compete with fluoropyrimidines for base‐nonspecific pyrimidine enzymes in cancer cells, thereby altering their metabolic flux for maximum efficacy. We demonstrate that the co‐delivery of CMP and FdUMP via FC‐NPs is well tolerated and more efficient than FdUMP alone in colorectal cancer models, and provide evidence of strong synergy with ICB immunotherapy that may improve current anticancer treatments.

## 4 Experimental Section

### 4.1 Cell Lines

CT26, MC38, MC38‐OVA, T24, 4T1, B16F10, A549, SW480, G422, HeLa, HT29, MCF‐7, DU145, and HCT116 cells obtained from ATCC, authenticated by STR profiling, and tested negative for mycoplasma contamination. 4T1, B16F10, A549, SW480, G422, HeLa, HT29, MCF‐7, DU145 and HCT116 cells were cultured in high glucose DMEM (Gibco) supplemented with 10% fetal bovine serum (FBS) (Gibco), 100 U/mL penicillin, 100 µg/mL streptomycin and 50 µg/mL gentamicin (Solarbio) at 37°C and 5% CO_2_ in a humidified atmosphere. CT26, MC38, MC38‐OVA and T24 cells were cultured under the same culture conditions as above except that the medium was replaced with RPMI‐1640 (Gibco). All the cell lines were mycoplasma contamination‐negative.

### 4.2 Mice

All animal studies were performed according to protocols approved by the Institute of Animal Care and Use Committee of Nanjing University. C57BL/6 and BALB/c mice of aged 6 to 8 weeks were purchased from the Comparative Medicine Centre of Yangzhou University and housed according to local guidelines. C57BL/6 *Sting1* ^−/−^ mice and C57BL/6 *Batf3* ^−/−^ mice were purchased from GemPharmatech, and 6‐ to 8‐week‐old mice were bred for experiments.

### 4.3 Gene Knockout Using Dual sgRNAs

For generation of stable Cas9‐expressing CT26 (CT26‐Cas9) cells. The Cas9 expression lentivirus (pLV[Exp]‐CBh>hCas9:T2A:Hygro) was purchased from VectorBuilder. CT26 cells were seeded in 6‐well plates (300 000 cells per well) and cultured for 12 h. An appropriate amount of lentivirus (multiplicity of infection, MOI = 20) was diluted into 1 mL of cell culture medium together with polybrene (5 µg/mL, VectorBuilder). The original medium was discarded, and the aforementioned lentivirus medium was added to the plate and incubated for 48 h. After incubation, lentivirus‐infected cells were selected using hygromycin (250 µg/mL) for 72 h.

Plasmid construction for knocking out CMPK1, CMPK2, UPP1, UPP2, and TYMP, respectively. sgRNAs were designed by using an online tool (https://design.synthego.com) and synthesized by GenScript. The pRP[2gRNA]‐Puro‐U6 vector was purchased from VectorBuilder. We used Mut Express II Fast Mutagenesis Kit V2 (Vazyme) to replace sgRNA sequences onto vectors to construct knockout plasmids. All sequences were verified by sequencing (Tsingke Biotechnology). CT26‐Cas9 cells were seeded into 6‐well plates and 24 h later. Transfection was performed on CT26‐Cas9 cells using 4 µg of knockout plasmid and lip8000 transfection reagent (Beyotime) according to the manufacturer's protocol. Successful and stable plasmid integration was maintained by culturing the cells in complete medium supplemented with 10 µg/mL puromycin (Beyotime). All sgRNA sequences are listed in Table S1. All vector information is listed in Table S2.

### 4.4 Negative‐Selection CRISPR‒Cas9‐Based Screen

Before the screen, the IC_50_ of FUDR in CT26‐Cas9 cells was determined in the same culture conditions of the screen. The sgRNA library was purchased from VectorBuilder, followed by CT26‐Cas9 transduction at an efficiency of 20% to avoid more than one sgRNA in each cell [37]. After the selection of transduced cells by puromycin (10 µg/mL) for 4 days, cells were divided into 2 replicates of each untreated and FUDR‐treated group. CT26‐Cas9 cells (4 × 10^7^) were collected on post‐transduction day 4 and day 11, from which genomic DNA was extracted by DNAzol (BioTeke) and sgRNA library deconvolution by HiSeq deep sequencing (VectorBuilder).

These data were analyzed by using the MAGeCK pipeline [38, 39]. Briefly, sequencing reads per sgRNA were aligned to the sgRNA library and the read counts of each sgRNA were calculated via MAGeCK. These read counts of each sgRNA were then normalized, and *p* values and false discovery rates (FDRs) were calculated for the depletion of each guide RNA on day 11 compared to day 4. Then, robust ranking aggregation (RRA) scores were calculated using the consistency of the behavior of all guide RNAs targeting the same gene to call negatively selected genes. Genes targeted by fewer than 4 distinct sgRNAs in the initial reference dataset and sgRNAs with fewer than 50 reads in the initial reference dataset were omitted during these analyses [24].

### 4.5 Cell Viability Assay

Cytotoxicity evaluation of different nucleobases, nucleosides, nucleotides and chemotherapeutics, as well as the combination of nucleobases, nucleosides, nucleotides and chemotherapeutics to cancer cells were evaluated by CCK‐8 (Dojindo Laboratories) assay. Cancer cells were seeded on 96‐well plates at 3 × 10^3^ per well and further cultured for 12 h. Then, the indicated drugs were incubated with cancer cells for 48 h—96 h before the CCK‐8 assay. To evaluate the synergistic effect of nucleobases, nucleosides and nucleotides with chemotherapeutics, we first obtained the IC_50_ of different chemotherapeutic drugs and subsequently evaluated the synergistic effect of these drugs by using a specified concentration of specified drug combined with different concentrations of the other specified drugs.

### 4.6 Western Blot Analyses

Cells were lysed by RIPA lysis buffer (Sigma Aldrich) with protease inhibitors and phosphatase inhibitors (Solarbio). The supernatants were collected, and the concentration of protein samples was quantified via a BCA Protein Assay Kit (Vazyme). The samples were boiled for 5 min, and each sample (10–20 µg) was subjected to 10%–12% SDS‐polyacrylamide gel electrophoresis (Meilunbio), and then transferred to polyvinylidene difluoride (PVDF) membranes (Millipore). The membranes were blocked with 5% non‐fat milk in TBS/Tween‐20 (0.05%) blocking buffer for 2 h at room temperature and then incubated with primary antibody solutions (TYMS 1:1000, SOD1 1:1000, STING 1:1000, cGAS 1:1000, p‐STING 1:1000, TBK1 1:1000, p‐TBK1 1:1000, IRF3 1:1000, p‐IRF3 1:1000, TYMP 1:200, UPP1 1:1000, UPP2 1:1000, β‐Actin 1:1000, CMPK1 1:1000, CMPK2 1:1000, VDAC1 1:1000) overnight at 4°C. Anti‐rabbit lgG HRP (1:3000) was used for further incubation with the membranes at room temperature for 1 h. Signals were detected using ECL Western Blotting Substrate (Tanon). β‐Actin or SOD1 was used as the control. Antibodies are listed in Table S3.

### 4.7 CETSA

For cellular level CETSA experiments [28], cells were inoculated in 6‐well plates (500 000 cells per well), treated with different test compounds and incubated for 2 h. Then, the cells were digested, washed once with PBS, resuspended in 1× PBS plus protease inhibitor (100 µL per well) and transferred to PCR tubes. The PCR tubes were incubated at 37°C for 3 min, then heated at the indicated temperatures for 3 min in a VeritiPro 96‐well thermal cycler and subsequently cooled down to 25°C and incubated for 3 min. After incubation, heat‐treated cell suspensions were quickly frozen in liquid nitrogen, and this process was repeated twice. Cell lysates were then centrifuged at 20 000 g and 4°C for 20 min to remove cellular debris and insoluble proteins. The supernatant was prepared for western blot analysis according to standard procedures. The apparent melting curve of TYMS was obtained by the method of CTSEA in the Information.

### 4.8 DARTS

A549 cells were treated with CMP or UMP (10 mM) for 2 h and then lysed with M‐PER mammalian protein extraction reagent (Thermo Scientific) with protease and phosphatase inhibitor cocktail (Solarbio) [29]. Samples were centrifuged at 15 000 g and 4°C for 10 min to clear the lysate from the cellular debris. The cell lysate was divided into 5 parts on average and degraded by pronase from Streptomyces griseus (Roche) (4 µg/mL, 2 µg/mL, 1 µg/mL, 0.5 µg/mL) for 30 min. Subsequently, 5× protein loading buffer (Solarbio) was added to the sample and incubated at 95°C for 5 min. Proteins were analyzed by western blotting according to standard procedures.

### 4.9 Quantification of Cytosolic Leakage of mtDNA

To obtain cytosolic and nuclear components of cells for determining cytosolic mtDNA, CT26 cells were pretreated in vitro with CMP (1 mM), FUDR (500 nM) or their combinations respectively for 24 h. Then, the cells were collected, and cytoplasmic components and other components of cells were extracted by the Cell Fractionation Kit (Abcam, ab109718) according to the manufacturer's guidelines. qPCR was used to determine the cytosolic leakage of mtDNA.

### 4.10 MtDNA Depletion

CT26 cells were treated with 20 µM 2’,3’‐dideoxycytidine (ddC, Aladdin) for 9 days [6].Then total DNA was extracted and qPCR was used to verify successful depletion of mtDNA. The copy number of mtDNA was normalized to that of nuclear DNA as the ratio of mtDNA encoding *CO1* and *Dloop1* to nuclear DNA encoding *Actb*. All primers are listed in Table S4.

### 4.11 Quantitative Real‐Time RT‒PCR Analysis

For qRT‒PCR, total RNA was isolated using the RNA‐easy isolation reagent (Vazyme). cDNA was reverse transcribed by TransScript IV One‐Step gDNA Removal and cDNA Synthesis SuperMix (TransGen) and was amplified with the TransStart Tip Green qPCR SuperMix (+Dye II) (TransGen). The sequences of the primers for targeted genes were synthesized by Genewiz and GenScript. Reactions were conducted using the QuantStudio 5 or Viia 7 Real‐Time PCR System (Applied Biosystems), and the data were analyzed with the QuantStudio Design and Analysis Software. Actb was used as an endogenous control, and differences between samples were calculated using the 2^−ΔΔCt^ (delta CT) method. All primers are listed in Table S4.

To detect the expression of *Ifnb1* with cGAS or STING inhibition, two inhibitors, RU.521 (MedChemExpress) and H‐151 (MedChemExpress), were used to inhibit cGAS and STING, respectively, where the inhibitors were incubated with cells for 2 h before drug treatment (H‐151 2 µM, RU.521 2 µM). dsDNA (500 ng) and DMXAA (Macklin,10 µg/mL) treated cells were used as positive controls. The dsDNA synthesized by Sangon Biotech, and the sequence is shown in Table S5.

### 4.12 Microscopy

For cell viability characterization of CMP+FUDR, A549 cells were seeded onto 24 well plates and treated with CMP (1 mM), FUDR (250 nM), TMP (100 µM) or their combination for 4 days. For cell viability characterization of the chemotherapeutics screen with CMP, A549 cells were treated with CMP (1 mM) and the indicated chemotherapeutics or their combination for 4 days. Cells were imaged using an Olympus CKX53 inverted microscope at a 40× or 100× magnification.

For cytosolic micronuclei detection, CT26 cells were seeded onto 15 mm confocal petri dishes (Biosharp) and treated with CMP (1 mM), FUDR (250 nM) or their combination for 16 h. For PicoGreen and DAPI staining, cells or tumor tissue sections were incubated with PicoGreen dsDNA Quantitation Reagent (Yeasen, 1:200, 10 min, 37°C), followed by washed with PBS and incubated with Antifade reagent with DAPI (Beyotime) [34]. Cells were imaged using an Olympus FV3000 confocal microscope at an oil immersion lens of 60× magnification. For cytosolic micronuclei area characterization, micronuclei areas were classified manually by distinct staining by PicoGreen of structures outside of the main nucleus.

### 4.13 LC‒MS/MS Analysis

For cellular and tumor tissue metabolite extraction. After being treated with specified drugs for the indicated different time intervals, cells in 6‐well plates were washed twice with 500 µL cold PBS and extracted with 500 µL −80°C pre‐cold extraction solution (80:20 v/v methanol/water) for 1 min on ice under agitation. For tumor tissue samples, harvested tumor tissues were homogenized thoroughly in the same pre‐cooled extraction solvent on ice prior to metabolite isolation. Then the supernatant was collected by centrifugation at 14 000 g for 10 min to obtain intracellular and intratumoral metabolites. Supernatants were transferred to fresh tubes and dried down using a speed vac concentrator (Labconco). Samples were resuspended in 100 µL of LC/MS‐grade H_2_O (Fisher Chemical).

Metabolites were analyzed using a Shimadzu triple quadrupole LC/MS/MS system LCMS‐8030 (LC‒MS/MS). First, the MS was operated in negative ionization mode monitoring the charge‐mass ratios (m/z) range 50–1000. The fragment m/z peaks of FdUMP, FdUTP, dUMP, TMP, uridine, and 2’‐deoxyuridine in LC‒MS/MS detection were screened and obtained as FdUMP (325.20>129.10 CE:23.0, 325.20>195.10 CE:15.0), FdUTP (484.80>404.85 CE:15.0, 484.80>387.00 CE:15.0, 484.80>159.10 CE:25.0, 484.0>79.00 CE:45.0), dUMP (307.20>195.05 CE:15.0), and TMP (321.20>195.05 CE:18.0, 321.20>125.10 CE:23.0), uridine (245.15>113.02 CE:18.0), 2’‐deoxyuridine (273.20>117.1 CE:18.0). The chromatographic conditions used were as follows: an Agilent ZORBAX SB‐CN (4.6 × 150 mm, 5 µm) chromatographic column was used; the mobile phase (A) was 0.1% acetic acid, and the mobile phase (B) was acetonitrile. The LC gradient program was as follows: 5% B at 0.01 min; 5%–20% B at 0.01–15 min; 20%–60% B at 15–18 min; 60%–5% B at 18–21 min; and 5% B at 21–30 min. The chromatographic column was kept at 40°C, 2 µL of the sample was injected, the flow rate was 0.4 mL/min, and the components within 7–15 min were collected for mass spectrometry analysis.

### 4.14 Synthesis of FC‐NPs, C‐NPs, FU‐NPs and U‐NPs

FC‐NPs, C‐NPs, FU‐NPs and U‐NPs were prepared via a water‐in‐oil reverse microemulsion method followed by thin‐film hydration [31, 32]. Briefly, two water‐in‐oil reverse microemulsions (10 mL) dispersed in a cyclohexane/IGEPAL CO‐520 (71:29, v/v) oil phase, were prepared. Emulsion 1 contained 500 µL FeCl_3_ solution (1 M) with 0.652 mg FdUMP, while emulsion 2 contained 500 µL CMP or UMP solution (2 M, pH 9) with 3 mg DOPA. The two emulsions were mixed and stirred for 30 min. Ethanol (50 mL) was added to remove the oil phase, and the phosphate cores were collected by centrifugation at 12 000 g for 20 min. After being carefully washed with ethanol three times, the phosphate cores together with DOPC (4 mg) and cholesterol (2 mg) were dissolved in chloroform. After the chloroform was removed by a rotary evaporator, the lipid film was then rehydrated in 500 µL Tris‐HCl buffer (10 mM, pH 7.4) to obtain FC‐NPs and FU‐NPs. C‐NPs and U‐NPs were prepared with the aforementioned processes by removing the FdUMP solution.

### 4.15 Characterization FC‐NPs and FU‐NPs

The hydrodynamic sizes of FC‐NPs and FU‐NPs were measured by dynamic light scattering (NanoBrook 90Plus, Brookhaven Instruments) at a concentration of 0.1 mg/mL. The morphologies of the NPs were visualized by transmission electron microscopy with sodium phosphotungstate staining. All images were acquired on an FEI Tecnai 20. To evaluate the drug loading efficiency and loading content of the FC‐NPs and FU‐NPs, FC‐NPs or FU‐NPs were dissolved in 0.1 M HCl to release FdUMP and CMP or UMP. The concentration of FdUMP was determined using LC‒MS/MS. While CMP and UMP were determined via measuring UV absorbance at 260 nm.

The in vitro release kinetics of CMP, UMP and FdUMP from the FC‐NPs and FU‐NPs under different pH conditions were determined by a dialysis method. 2 mL of FC‐NPs and FU‐NPs were sealed in dialysis bags (molecular weight cut‐off of 1 kD) respectively and immersed in 50 mL of buffer with different pH values (pH 5.5, 6.5, 7.4). The dialysis systems were incubated in a 37°C magnetic stirrer system, and 100 µL samples outside the dialysis bag were collected at different time intervals for CMP, UMP and FdUMP quantification. An equal volume of prewarmed fresh buffer was added back to the dialysis systems.

For cellular uptake analysis, FC‐NPs and FU‐NPs were prepared with the aforementioned processes by adding 1,1’‐dioctadecyl‐3,3,3’,3’‐tetramethylindodicarbocyanine, 4‐chlorobenzenesulfonate salt (DiD), a far‐red plasma membrane fluorescent probe for labelling lipid layers. Then they incubated with CT26 cells for the indicated different time intervals, followed by analysis with flow cytometry (CytExpert 4.5, Beckman Coulter).

### 4.16 In Vivo Immune Response Analysis

OVA antigen‐specific T cells in PBMCs and spleen were analyzed. Briefly, the PBMCs and spleens were collected from MC38 xenograft mice. For PBMC analysis, 10 µL of T‐Select MHC Tetramer‐APC (MBL) was added to 200 µL of whole blood sample. The mixture was gently vortexed, and then incubated at 4°C for 30 min. Subsequently, anti‐CD45.2‐FITC, anti‐CD3‐PE/Cyanine7, and anti‐CD8α‐PE antibodies were added and incubated at 4°C for 30 min. Then, these samples were hemolyzed using ACK red blood cell lysate (Solarbio), followed by fixation with 0.5% paraformaldehyde fixative (Servicebio). The cells were resuspended in 500 µL of PBS, followed by analysis with flow cytometry. For spleen T cells analysis, the isolated spleen was ground and passed through a 100‐mesh cell sieve to collect the single‐cell suspension. The single‐cell suspension was washed twice with pre‐cooled PBS and blocked with CD16/32 antibody for 10 min. Then, the cells were stained with the aforementioned processes, followed by analysis with flow cytometry. All the flow cytometric analyses were performed using a Cytoflex S and analyzed using CytExpert 4.5 (Beckman Coulter). Antibodies and T‐Select MHC Tetramer‐APC are listed in Table S3.

ELISA for the detection of cytokines. At the indicated time points, the cytokine levels of IFN‐β (Biolegend), TNF‐α, CXCL‐10 and CCL‐5 in serum were measured by ELISA (ABclonal). For IFN‐γ in tumor quantification, tumors were isolated at the indicated time points and homogenized with a homogenizer in an ice‐bath (IKA). Suspensions were centrifuged at 3500 g for 15 min, and the supernatants were collected for ELISA assay. All ELISA kits were purchased from ABclonal except for IFN‐β (Biolegend).

### 4.17 In Vivo Therapeutic Study

Therapeutic studies were carried out in CT26 and MC38 xenograft models. For the CT26 xenografts, female BALB/c mice of aged 6–8 weeks were subcutaneously injected with 100 µL of a single‐cell suspension of 5 × 10^5^ CT26 colon cancer cells in the right back flank. For the subcutaneous MC38 xenografts and MC38‐OVA xenografts, female C57BL/6 mice, *Sting1* ^−/−^ C57BL/6 mice or *Batf3* ^−/−^ C57BL/6 mice were subcutaneously injected with 100 µL of a single‐cell suspension of 5 × 10^5^ MC38 colon cancer cells or MC38‐OVA colon cancer cells in the right back flank. All animal experiments were performed according to protocols approved by the Institute of Animal Care and Use Committee of the Nanjing University. Tumor‐bearing mice were divided randomly into different groups with approximately equal tumor volumes. The indicated drugs or formulations were administered by the indicated route at the indicated time points as shown in related therapy schedule figures. For tumor volume measurements, two perpendicular diameters of the tumor were measured by a caliper, and the tumor volumes were calculated using the formula volume = 0.5 × A × B^2^, where A and B are the larger and smaller diameters, respectively. The differences in survival in each group were determined using the Kaplan–Meier method, and the overall *p* value was calculated by the log‐rank test using GraphPad Prism. Animals were euthanized when the tumor reached 2000 mm^3^ in size or when they became moribund with severe weight loss or unhealing ulceration. At the indicated time points, the cytokine levels in serum and tumors were assessed by using ELISA as described above. Meanwhile, the percentages of tumor antigen‐specific CD8α+ T cells among PBMCs and spleen were analyzed as described above for the OVA peptide–major histocompatibility complex (MHC) tetramer. The antibodies used in vivo are listed in Table S3.

### 4.18 Statistical Analysis

All data in the present study are expressed as the mean ± s.d. unless indicated in the figure legends. Statistical analysis was performed using GraphPad Prism 9.3.1 (GraphPad Software). The significance between two groups was analyzed by a two‐tailed Student's *t*‐test. Sample variance was tested using the F‐test. For multiple comparisons, a one‐way or a two‐way ANOVA test was used. In all cases, a *p* value less than 0.05 was considered significant. No samples were excluded from the analysis.

## Author Contributions

Conceptualization: C. Wang, J. Wu; Data curation: C. Wang, C. Zhao, J. Wu; Formal analysis: C. Wang, J. Wu; Funding acquisition: J. Wu, C. Wang; Methodology: C. Wang, Y. Liu, J. Wu; Experiments: C. Wang, C. Zhao, Y. Yao, Z. Zou, A. Shi, Y. Liu, Y. Yu, Z. Feng; Project administration: J. Wu, Y. Hu; Resources: C. Wang, C. Zhao, Z. Zou, A. Shi; Software: C. Wang, C. Zhao; Supervision: J. Wu, X. Rui; Validation: C. Wang, J. Wu; Visualization: C. Wang, C. Zhao; Writing – original draft: C. Wang, C. Zhao; Writing – review & editing: C. Wang, C. Zhao, J. Wu, X. Rui.

## Ethics Statement

This research complied with all relevant ethical regulations for animal experimentation. The experimental protocol was reviewed and approved by the Institute of Animal Care and Use Committee of the Nanjing University (IACUC‐2003160). All efforts were made to minimize animal suffering and reduce the number of animals used.

## Conflicts of Interest

J. Wu., C. Wang, C. Zhao, and Y. Yao are authors of a patent related to this work (CN202411691345.6) filed by Nanjing University, China. The other authors declare no competing interests.

## Supplementary Information

Methods and Materials, Tables S1–S5 and Figures S1–S35.

## Supporting information




**Supporting File**: advs77614‐sup‐0001‐SuppMat.docx.

## Data Availability

The authors declare that data supporting the findings of this study are available within the article and its Information files. All relevant data are available from the corresponding authors upon reasonable request.
