# Supplementary material for: Ribonucleotides Amplify Mitochondrial DNA‐Driven cGAS–STING Activation via FdUMP‐ribonucleotide Hybrid Particles to Potentiate Chemoimmunotherapy
Source: Adv Sci (Weinh). 2026 Sep 21:e77614. Online ahead of print. doi: 10.1002/advs.77614 (PMC13591344; doi:10.1002/advs.77614)
Supplement: Supplementary file 1 [file ADVS-9999-e77614-s001.docx]

Supplementary Materials for

**Ribonucleotides amplify mitochondrial DNA-driven cGAS-STING activation via FdUMP-ribonucleotide hybrid particles to potentiate chemoimmunotherapy**

Chao Wang^1,5^†*, Chenxuan Zhao^1^†, Yesong Yao^1^, Yang Liu^1^, Zhiwen Zou^1^, Anxin Shi^1^, Zhuo Feng^1^, Mingkang Yang^1^, Yue Yu^1^, Xuehui Rui^1,2,3,^*, Jinhui Wu^1,2,3,4^*

Correspondence to: wuj@nju.edu.cn (J.W.); xuehui.rui@outlook.com (X.R.) and nan.wangchao@qq.com (C.W.).

**This file includes:**

Supplementary Figures. 1 to 35

Tables S1 to S5

Supplementary methods

Supplementary reference

**Figures. S1 to S31**

**Supplementary Fig. 1.** CT26 cells were treated with ddC for 9 days to inhibit mtDNA synthesis, then these cells and control CT26 cells were treated in vitro with CMP, FUDR, TMP or their combinations respectively for 24 h. The expression of *Ifnb1* and *Mx1* was determined by qRT‒PCR (*n* = 3).

**Supplementary Fig. 2**. **a**, qPCR analysis of mtDNA copy number in mitochondrial fractions of CT26 tumor cells treated with CMP+FUDR and corresponding control regimens for 24 h (n = 3). A schematic of the mtDNA and of the used genes in the assay is shown at the bottom right. The blue dashed line represents the major arc, and the remaining part of mtDNA is minor arc. **b**, CT26 cells were preincubated with VBIT-4 or vehicle DMSO for 8 h, followed by 24 h treatment with CMP+FUDR and corresponding control regimens. Relative *Ifnb1* transcript levels were quantified via qRT-PCR (n = 6). **c**, Western blotting was performed on total and cytosolic protein fractions from CT26 cells using anti-β-Actin and anti-VDAC1 antibodies. Error bars represent SEM for all panels, and centre values represent the mean. Statistical significance was assessed using one-way ANOVA (**a**) and a two-tailed Student’s t-test (**b**).

**Supplementary Fig. 3.** CT26 cells were treated in vitro with FUDR or with of gradient concentrations TMP plus FUDR (4 μM) for 24 h. Expression of *Ifnb1* was determined by qRT-PCR (*n* = 3).

**Supplementary Fig. 4. a**, Schematics depicting the therapeutic schedule of MC38 cancer xenografts STING-deficient (*Sting1*^-/-^) C57BL/6 mice and *Sting1*^+/+^ mice treated with intratumoral injection of CMP+FUDR. **b**, MC38 tumor weight in (**a**). These mice were treated with Saline (*n* = 5), CMP (*n* = 5), FUDR (*n* = 5) or CMP+FUDR (*n* = 5). MC38 tumor weight at the endpoint day 24 with each dot representing a mouse. Data information: Error bars represent SD for all panels, and centre values represent the mean. Statistical significance was assessed using one-way ANOVA (**b**).

**Supplementary Fig. 5.** a. In The CRISPR–Cas9-based gRNA library negative screen, gRNA reads in CT26-Cas9 cells after FUDR treatment at day 7 was compared to day 0. Lines of different colours represent multiple gRNAs corresponding to the same gene.

**Supplementary Fig. 6.** DARTS analysis of CMPK1, CMPK2, TYMP, UPP1 and UPP2 stabilization in A549 lysates that were incubated with 1mM UMP or vehicle for 30 min, followed by protein digestion at increasing pronase concentration and assessment of CMPK1, CMPK2,TYMP, UPP1 and UPP2 degradation by western blot.

**Supplementary Fig. 7. a**.Western blot for WT CT26 cells, CMPK1-KO CT26 cells, CMPK2-KO CT26 cells, TYMP-KO CT26 cells, UPP1-KO CT26 cells and UPP2-KO CT26 cells, assayed with CMPK1, CMPK2, TYMP, UPP1, UPP2 and beta-Actin antibodies.

**Supplementary Fig. 8**. **a-c**, CT26 cells were treated with CMP+FUDR and corresponding control regimens for 1 h. Intracellular TMP (**a**), dUMP (**b**), and FdUMP (**c**) concentrations were quantified via LC-MS/MS (n = 6).

**Supplementary Fig. 9.** a. 57℃ CETSA analysis of TYMS stabilization in CT26 cells upon FTDR (50 nM) and gradient concentration of CMP treatment for 6 h. Protein levels were normalized against SOD1. b, 57℃ CETSA analysis of TYMS stabilization in CT26 cells upon FCDR (50 nM) and gradient concentration of CMP treatment for 6 h. Protein levels were normalized against SOD1

**Supplementary Fig. 10**. **a** and **b**, CT26 cells were treated with CMP+FUDR and corresponding control regimens for 1 h. Intracellular uridine (**a**) and 2'-deoxyuridine (**b**) concentrations were quantified via LC-MS/MS (n = 3).

**Supplementary Fig. 11.** LC-MS/MS for determining FdUMP, dUMP, FdUTP and TMP intensity of WT CT26 cells, *Cmpk1*-KO CT26 cells, *Cmpk2*-KO CT26 cells, *Tymp*-KO CT26 cells, *Upp1*-KO CT26 cells and *Upp2*-KO CT26 cells treated with FUDR (1 μM) or FUDR+CMP (0.25 mM) for 2 h.

**Supplementary Fig. 12.** CMP can enhance the therapeutic activity of FUDR in various tumor cell lines. CMP enhanced the therapeutic activity of FUDR against mouse melanoma B16F10 cells (a), mouse colon cancer MC38 cells (c), mouse breast cancer 4T1 cells (d), human colon cancer SW480 cells (e), human colon cancer HT29 cells (f), human colon cancer HCT116 cells (g), human prostate cancer DU145 cells (h), and human non-small cell lung cancer A549 cells (j).CMP slightly increased the therapeutic activity of FUDR on mouse glioma G422 cells (b), human breast cancer MCF7 cells (i), humancervical cancer Hela cells (k), and human bladder cancer T24 cells (l). The top half of Figures a-l shows the growth inhibitory concentration profiles of FUDR on each cell line, and the bottom half of the Figures a-l show the growth inhibitory concentration profiles of FUDR at indicated concentrations in combination with gradient concentrations of CMP on each cell line.

**Supplementary Fig. 13.** **a**, CT26 tumor weight at the endpoint from (**d**), with each dot representing a mouse. **b**, Pictures of CT26tumorsand spleens at the endpoint day 22. **c**, Spleen weight at the endpoint day 22, with each dot representing a mouse. **d**, Body weight curve of control BALB/c mice (*n* = 9), BALB/c mice treated with CMP (*n* = 9), BALB/c mice treated with FdUMP (*n* = 9), or BALB/c mice treated with FdUMP+CMP (*n* = 9).

**Supplementary Fig. 14.** a, Self-assembly behaviour of CMP, FdUMP and Fe^3+^. Transmission electron microscopy (TEM) images of CMP-Fe or FdUMP-Fe coordination polymers formed by mixing CMP and FdUMP with Fe^3+^ (4:1, n/n) for 1 h. Scale bars, 200 nm; n/n, molar ratio. b, Schematic illustration of FCMPs structure. b, Self-assembly behaviour of UMP, FdUMP and Fe^3+^ . Transmission electron microscopy (TEM) images of UMP-Fe or FdUMP-Fe coordination polymers formed by mixing UMP, FdUMP with Fe^3+^ (2:1, n/n) for 1 h. n/n, molar ratio.

**Supplementary Fig. 15.** TEM images showing homogeneous FU-NPs formed by

coating FdUMP/UMP–Fe coordination polymers with a PEGylated lipid layer.

**Supplementary Fig. 16.** Dynamic light scattering analyses of FU-NPs.

**Supplementary Fig. 17.** The UMP (left) and FdUMP (right) release profiles from FU-NPs under different pH values.

**Supplementary Fig. 18.** FU-NPs increased the cellular uptake of UMP and FdUMP at the same time. CT26 cells were incubated with FU-NPs for 2, 4, 8, 12 or 24 h, followed by analysis by flow cytometry.

**Supplementary Fig. 19.** Relative viability of CT26 cells treated with C-NPs+250 nM FdUMP for 48 h.

**Supplementary Fig. 20.** Relative viability of CT26 cells treated with FU-NPs or U-NPs+250 nM FUDR for 48 h.

**Supplementary Fig. 21.** CT26 cells were treated in vitro with FUDR (250 nM), UMP (1 mM), UMP+FUDR, U-NPs, U-NPs+FUDR, FU-NPs or untreated control for 24 h. The expression of *Ifnb1* was determined by qRT–PCR (*n* = 3).

Data information: Error bars represent SD for all panels, and centre values represent the mean. Statistical significance was assessed using one-way ANOVA.

**Supplementary Fig. 22.** Schematics depicting the therapeutic schedule and immunoassay of MC38 cancer xenografts treated with intratumoral injection of FdUMP+UMP or FU-NPs.

**Supplementary Fig. 23.** FU-NPs treatment significantly increased the secretion of inflammatory cytokines. Serum cytokines (IFN-β, TNF-α, CXCL-10, CCL-5) and intratumoral IFN-γ were measured by ELISA on day 14 and day 21 after the third dose, respectively. Data information: Error bars represent SEM for all panels, and centre values represent the mean. Statistical significance was assessed using one-way ANOVA.

**Supplementary Fig. 24. a**, Growth curves of MC38 tumors in individual mice treated with intratumoral injection of FdUMP+UMP or FU-NPs or untreated. **b**, FU-NPs inhibited the growth of MC38 tumors and prolonged the survival of mice. Average tumor-growth curves of MC38 tumors and survival curves of the mice in (**a**) (n = 7). Data information: Error bars represent SEM for all panels, and centre values represent the mean. Statistical significance was assessed using two-way ANOVA (**b**) with Bonferroni’s multiple comparisons test, or log-rank (Mantel‒Cox) test (**b**).

**Supplementary Fig. 25.** Schematics depicting the therapeutic schedule of CT26 cancer xenografts treated with intratumoral injection of FdUMP+UMP or FU-NPs.

**Supplementary Fig. 26. a**, Growth curves of CT26tumorsin individual mice treated with intratumoral injection of FdUMP+UMP or FU-NPs or untreated (*n* = 7). **b**, FU-NPs inhibited the growth of CT26 tumors. Average tumor-growth curves of CT26 tumors of the mice in (**a**). Data information: Error bars represent SEM for all panels, and centre values represent the mean. Statistical significance was assessed using two-way ANOVA (**b**).

**Supplementary Fig. 27.** Schematics depicting the therapeutic schedule, T cell analysis and immunoassay of MC38-OVA cancer xenografts treated with intratumoral injection of FdUMP+UMP or FU-NPs.

**Supplementary Fig. 28. a**, The ratio of antigen-specific T cells in PBMCs was significantly increased after FU-NPs treatment. Representative scatter plots for OVA tetramer+ CD8 T cells and statistical analysis of the OVA tetramer+ CD8 T cells in peripheral blood T cells. The antigen-specific T cell response in PBMCs was analysed by OVA tetramer staining on day 21. **b**, Interferon-γ was increased in MC38-OVA tumors after FU-NPs treatment. Intratumoral IFN-γ was measured by ELISA on day 21 after the third dose. Data information: Error bars represent SEM for all panels, and centre values represent the mean. Statistical significance was assessed using one-way ANOVA.

**Supplementary Fig. 29. a**, Schematics depicting the therapeutic schedule of MC38 cancer xenografts in *Batf3*^-/-^ mice and *Batf3*^+/+^ mice treated with intratumoral injection of FU-NPs or untreated. **b**, The therapeutic effect of FU-NPs on MC38 tumors was impaired in *Batf3*^-/-^ mice. Average tumor-growth curves of MC38tumorsof the mice in (**a**) (*n* = 8). Data information: Error bars represent SEM for all panels, and centre values represent the mean. Statistical significance was assessed using two-way ANOVA (**b**).

**Supplementary Fig. 30. a**, Schematics depicting the therapeutic schedule of MC38 cancer xenografts treated with intraperitoneal injection of anti-CD8α antibody (aCD8) or isotype control antibody (Isotype) and intratumoral injection of FU-NPs. **b**, The therapeutic effect of FU-NPs on MC38 tumors was severely impaired by CD8+ T cell depletion. Average tumor-growth curves of MC38tumorsof the mice in (**m**) (*n* = 8). Data information: Error bars represent SEM for all panels, and centre values represent the mean. Statistical significance was assessed using two-way ANOVA (**b**).

**Supplementary Fig. 31. a**, The therapeutic effect of anti-PD-L1 on MC38tumorswas potentiated by FU-NPs treatment. Growth curves of MC38tumorsin individual mice in (**c**) (*n* = 8). **b**, The combination of anti-PD-L1+FU-NPs caused mice to completely reject MC38tumorsand prolonged the survival of mice. Survival curves of the mice in (**c**). **c**, Schematics depicting the therapeutic schedule of MC38 cancer xenografts treated with intraperitoneal injection of anti-PD-L1 antibody (aPD-L1) or isotype control antibody (Isotype) and a combination of intratumoral injection of FU-NPs. Data information: Error bars represent SEM for all panels, and centre values represent the mean. Statistical significance was assessed using Bonferroni’s multiple comparisons test, or log-rank (Mantel‒Cox) test (**b**).

**Supplementary Fig. 32. CMP augments the therapeutic activity of pseudopyrimidine chemotherapeutics.** **a**, Chemical structures of CMP and pseudopyrimidine chemotherapeutics. **b**, Relative viability of CT26 cells treated with pseudopyrimidine chemotherapeutics in the presence of CMP at gradient concentrations versus CMP control (n = 3).

**Supplementary Fig. 33.** **dUMP augments the therapeutic activity of pseudopyrimidine chemotherapeutics. a**, Chemical structures of dUMP, gemcitabine, decitabine and Ara-C. **b**, dUMP enhances the therapeutic activity of nucleotide drugs targeting CMP and dCMP metabolism. Relative viability of CT26 cells treated with gemcitabine (32 nM), decitabine (8 μM) and Ara-C (3 μM) in the presence of dUMP at gradient concentrations versus dUMP control (*n* = 3). Ara-C, Cytarabine.

**Supplementary Fig. 34.** Relative viability of CT26 cells treated with CMP, CMP+Gemcitabine (32 nM).

**Supplementary Fig. 35**. **a**, H&E-stained tissue sections of major organs including liver, heart, spleen, lung, and kidney of mice receiving i.t. injection of CMP (500 mg/kg). Those mice were examined at day 2 and day 28 post injection. Saline treated mice were used as the control. (For liver, spleen, and heart, scale bar=100μm; for kidney, scale bar =500μm; for lung, scale bar =1 mm). **b**, Blood biochemistry and complete blood analysis of mice receiving i.t. injection of saline or CMP (500 mg/kg) at day 2 and day 28 post injection. The examined parameters included alanine aminotransferase (ALT), aspartate aminotransferase (AST), alkaline phosphatase (ALP), blood urea nitrogen (BUN), creatinine (CR), lymphocyte counts, mean corpuscular volume (MCV), hematocrit (HCT), hemoglobin (HGB), neutrophil count(Neu), red blood cell count (RBC) and white blood cell count (WBC), (n = 3).

**Tables S1 to S5**

**TableS1. sgRNA sequences in plasmid**

| **Plasmid** | **Target** | **sgRNA sequences 1** | | **sgRNA sequences 2** |
| --- | --- | --- | --- | --- |
| pRP[2gRNA]-Puro-U6-sgCmpk1 | CPMK1 | | AGCTCGGGACCAGGACGTGC | AGTTCACCATACTGTGAGTC |
| pRP[2gRNA]-Puro-U6-sgCmpk2 | CMPK2 | | GGGCGGCTTATTAGGGCCAT | GTGCCCCTGGCTCCAGGCGA |
| pRP[2gRNA]-Puro-U6-sgUpp1 | UPP1 | | AGTGAGAACATGGCAGCTAC | CTATGTTTGGATTTGAAAGT |
| pRP[2gRNA]-Puro-U6-sgUpp2 | UPP2 | | TGACAAGAATACATACGAGAGG | CGAAGACATTCTCTATCACTTGG |
| pRP[2gRNA]-Puro-U6-sgTymp | TYMP | | TCCAGGGACACCACCTCCCT | GGTGATAGATGGAAGAGCAC |

**Table S2. Vector information.**

| **Plasmid and Lentivirus** | **Source** | **Identifier** |
| --- | --- | --- |
| pLV[Exp]-CBh>hCas9:T2A:Hygro | VectorBuilder | Cat# LVS (B160923-1033trt) |
| Mouse whole-genome dual-gRNA lentivirus library | VectorBuilder | Cat# LVM (lib190505-1050kpm) |
| pRP[2gRNA]-Puro-U6-sg*Cmpk1* | VectorBuilder | Cat# Ecoli (VB230206-1164uga) |
| pRP[2gRNA]-Puro-U6-sg*Cmpk2* | VectorBuilder | Cat# Ecoli (VB230206-1145gve) |
| pRP[2gRNA]-Puro-U6-sg*Upp1* | This paper | N/A |
| pRP[2gRNA]-Puro-U6-sg*Upp2* | This paper | N/A |
| pRP[2gRNA]-Puro-U6-sg*Tymp* | This paper | N/A |
| pLVML-3×FLAG-*Cmpk1*-Puro | This paper | N/A |
| pLVML-3×FLAG-*Cmpk2*-Puro | This paper | N/A |

**Table S3. Antibodies information.**

| **Antibodies** | **Source** | **Identifier** |
| --- | --- | --- |
| Rabbit anti-TYMS | Proteintech | Cat# 15047-1-AP |
| Rabbit anti-SOD1 | Proteintech | Cat# 10269-1-AP |
| Rabbit anti-Beta Actin | Proteintech | Cat# 20536-1-AP |
| Anti-rabbit lgG HRP | Proteintech | Cat# SA00001-2 |
| Rabbit anti-STING | Cell Signaling Technology | Cat# 50494 |
| Rabbit anti-cGAS | Cell Signaling Technology | Cat# 31659 |
| Rabbit anti-phospho-STING | Cell Signaling Technology | Cat# 72971 |
| Rabbit anti-TBK1/NAK | Cell Signaling Technology | Cat# 3504 |
| Rabbit anti-phospho-TBK1/NAK | Cell Signaling Technology | Cat# 5483 |
| Rabbit anti-IRF-3 | Cell Signaling Technology | Cat# 4302 |
| Rabbit anti-phospho-IRF-3 | Cell Signaling Technology | Cat# 29047 |
| Rabbit anti-TYMP | ABclonal | Cat# A1094 |
| Rabbit anti-UPP1 | ABclonal | Cat# A0613 |
| Rabbit anti-UPP2 | Cusabio technology | Cat# CSB-PA025660LA01HU |
| Rabbit anti-UMP-CMP Kinase | Immunoway | Cat# YT4821 |
| Rabbit anti-CMPK2 | Immunoway | Cat# YT6811 |
| Anti-mouse CD16/32 antibody | Elabscience | Cat# E-AB-F0997 |
| Anti-mouse CD3 antibody | Elabscience | Cat# E-AB-F1013H |
| Anti-mouse CD45.2 antibody | Elabscience | Cat# E-AB-F1122C |
| Anti-mouse CD8α antibody | Elabscience | Cat# E-AB-F1104A |
| FITC Mouse IgG2a | Elabscience | Cat# E-AB-F09802C |
| PE Mouse IgG2a | Elabscience | Cat# E-AB-F09802D |
| PE/Cyanine7 Mouse IgG2a | Elabscience | Cat# E-AB-F09803H |
| T-Select H-2Kb OVA Tetramer-SIINFEKL-APC | MBL | Cat# TS-5001-2C |
| H-2Kb Negative Tetramer-SIYRYYGL-APC | MBL | Cat# TS-M008-2 |
| InVivoMAb Anti-mouse CD8α | Bio X Cell | Cat#:BE004-1 |
| InVivoMAb rat IgG2a isotype control | Bio X Cell | Cat#BE0089 |
| InVivoMAb Anti-mouse PD-L1(B7-H1) | Bio X Cell | Cat#BE0101 |
| InVivoMAb rat IgG2b isotype control | Bio X Cell | Cat#BE0090 |

**Table S4. Primer sequences for qRT-PCR and qPCR**

| **Gene** | **Forward primer 5’-3’** | **Reverse primer 5’-3’** |
| --- | --- | --- |
| *Ifnb1* | GCCTTTGCCATCCAAGAGATGC | ACACTGTCTGCTGGTGGAGTTC |
| *IFNB1* | CTTGGATTCCTACAAAGAAGCAGC | TCCTCCTTCTGGAACTGCTGCA |
| *Actb* | CATTGCTGACAGGATGCAGAAGG | TGCTGGAAGGTGGACAGTGAGG |
| *ACTB* | CACCATTGGCAATGAGCGGTTC | AGGTCTTTGCGGATGTCCACGT |
| *Cmpk1* | TGAAGCCGTTGGTCGTGTTCGT | GAAGCTCTCCTGCAGAAAGGTG |
| *CMPK1* | ATGAAGCCGCTGGTCGTGTTCG | CCTTTCATCACGAAGCAGCTCTC |
| *Cmpk2* | AACTCTGCGGTGTTCCAAGACC | GGAACTTCCCTTTCTGGACCTC |
| *CMPK2* | CCAGGTTGTTGCCATCGAAGGA | GGTGGTGACTTTAAGAGGACAGC |
| *Mx1* | GACCATAGGGGTCTTGACCAA | AGACTTGCTCTTTCTGAAAAGCC |
| *mtDloop* | AATCTACCATCCTCCGTGAAACC | TCAGTTTAGCTACCCCCAAGTTTAA |
| *mtCOX1* | GCCCCAGATATAGCATTCCC | GTTCATCCTGTTCCTGCTCC |
| *mtCytb* | GCTTTCCACTTCATCTTACCATTTA | TGTTGGGTTGTTTGATCCTG |
| *mtCOX3* | CCTCGTACCAACACACATGATCTAGG | AGTGGGACTTCTAGAGGGTTAAGT |
| *mtNd1* | GAGCCTCAAACTCCAAATACTCACT | GAACTGATAAAAGGATAATAGCTATGGTTACTTCA |
| *mtNd4* | GAAGCAACCTTAATCCCAACAC | AGCAGTGGAATAGAACCGATTAG |

**Table S5. dsDNA sequences**

| **DNA** | **Sequence** |
| --- | --- |
| dsDNA | 5-TACAGATCTACTAGTGATCTATGACTGATCTGTACATGATCTACA[1,2] |
| dsDNA | 5-TGTAGATCATGTACAGATCAGTCATAGATCACTAGTAGATCTGTA |

**Supplementary reference**

[1] Jiaxi Wu et al. ,Cyclic GMP-AMP Is an Endogenous Second Messenger in Innate Immune Signaling by Cytosolic DNA.Science339,826-830(2013).DOI:10.1126/science.1229963

[2] Lijun Sun et al. ,Cyclic GMP-AMP Synthase Is a Cytosolic DNA Sensor That Activates the Type I Interferon Pathway.Science339,786-791(2013).DOI:10.1126/science.1232458
